# A nuclear pore sub-complex restricts the propagation of Ty retrotransposons by limiting their transcription

**DOI:** 10.1371/journal.pgen.1009889

**Published:** 2021-11-01

**Authors:** Amandine Bonnet, Carole Chaput, Noé Palmic, Benoit Palancade, Pascale Lesage

**Affiliations:** 1 Université de Paris, Institut de Recherche Saint-Louis, INSERM U944, CNRS UMR 7212, Paris, France; 2 Université de Paris, CNRS, Institut Jacques Monod, Paris, France; Cornell University, UNITED STATES

## Abstract

Beyond their canonical function in nucleocytoplasmic exchanges, nuclear pore complexes (NPCs) regulate the expression of protein-coding genes. Here, we have implemented transcriptomic and molecular methods to specifically address the impact of the NPC on retroelements, which are present in multiple copies in genomes. We report a novel function for the Nup84 complex, a core NPC building block, in specifically restricting the transcription of LTR-retrotransposons in yeast. Nup84 complex-dependent repression impacts both *Copia* and *Gypsy* Ty LTR-retrotransposons, all over the *S*. *cerevisiae* genome. Mechanistically, the Nup84 complex restricts the transcription of Ty1, the most active yeast retrotransposon, through the tethering of the SUMO-deconjugating enzyme Ulp1 to NPCs. Strikingly, the modest accumulation of Ty1 RNAs caused by Nup84 complex loss-of-function is sufficient to trigger an important increase of Ty1 cDNA levels, resulting in massive Ty1 retrotransposition. Altogether, our study expands our understanding of the complex interactions between retrotransposons and the NPC, and highlights the importance for the cells to keep retrotransposons under tight transcriptional control.

## Author summary

Retroelements, which replicate by reverse transcription of their RNA into a cDNA that is integrated into the host genetic material, play an important role in the plasticity of eukaryotic genomes. The study of yeast retrotransposons has led to the identification of host factors that limit or facilitate retroelement mobility, including components of the nuclear pore complex (NPC), most of them still awaiting mechanistic characterization. Here, we investigated the contribution of the Nup84 complex, a core NPC scaffold, to retrotransposon biology in budding yeast. Our findings uncover that the Nup84 complex restricts the transcription of phylogenetically-distinct Ty retroelements. By focusing on Ty1 retrotransposons, we provide evidence that repression by the Nup84 complex depends on the maintenance at the NPC of the SUMO-protease Ulp1, an essential enzyme of the SUMO pathway with multiple targets in the transcription machinery. We finally show that this transcriptional control is critical for genome dynamics, since a small increase in the accumulation of Ty1 RNAs leads to massive retrotransposition. Our data reveal that although relatively abundant, Ty transcripts are limiting for retrotransposition, underscoring the importance of having their expression tightly controlled. We also characterize a new non-canonical function of NPCs, confirming their connection with genome expression and stability.

## Introduction

As the unique gates between the cytoplasm and the nucleus, nuclear pore complexes (NPCs) are key components of eukaryotic cells. NPCs are modular, megadalton-sized multiprotein assemblies built from multiple copies of ~30 distinct proteins called nucleoporins (Nups) and associated into sub-complexes (reviewed in [1,2]). The overall structural organization of the NPC is conserved across eukaryotes, with a core scaffold embedded within the nuclear envelope and peripheral components, namely the cytoplasmic filaments and the nuclear basket, extending towards the cytoplasm and the nucleoplasm, respectively.

Beyond their canonical role in the selective transport of proteins and RNAs, NPCs have also emerged as key platforms for the three-dimensional organization of the genome, thereby impacting the regulation of gene expression and the maintenance of genetic integrity (reviewed in [3–5]). Genome-wide approaches, together with the live imaging of individually-tagged loci, have revealed that nucleoporins associate mostly with transcriptionally-active genes, but also with repressed regions and chromatin boundaries, from yeasts to metazoans [6–14]. While a subset of nucleoporins can bind their target sequences away from the NPC [15–17], these interactions typically occur at the nuclear envelope, as exemplified by the well-described recruitment of induced genes to the pores in budding yeast [18–21]. Such functional associations between NPCs and the genome are relevant for the establishment of expression patterns, as revealed by the transcriptional changes triggered in *cis* of the Nup-interaction sites upon nucleoporin loss-of-function (reviewed in [5,22]). Although NPCs have been proposed to serve as scaffolds for transcriptional activities, impacting chromatin organization and mRNA biogenesis [5,20], the direct and indirect mechanisms underlying their function in gene expression remain to be fully elucidated.

While a critical role in transcription has been assigned to components of the nuclear pore basket, which are closest to the genome [11,23–26], gene expression has also been reported to depend on the core NPC scaffold [9,27,28], including the Y-complex (a.k.a. the Nup84 complex in budding yeast). This module comprises seven subunits in *S*. *cerevisiae* (Nup84, Nup85, Nup120, Nup133, Nup145C, Sec13, and Seh1) and inactivation of most of them displays a common phenotypic signature, likely reflecting their structural interdependence for stable NPC association [29,30]. Apart from its critical role in NPC distribution and mRNA export [29], the yeast Nup84 complex has been shown to participate to the maintenance of genome integrity [31,32] and to multiple steps in transcriptional regulation, including activation, repression and elongation [32–36]. In some cases, the Nup84 complex was shown to regulate these processes by tethering the SUMO-deconjugating enzyme Ulp1, an essential member of the SUMO modification pathway with multiple targets in the DNA and RNA metabolism machineries (reviewed in [37–39]), including transcription factors whose activity is locally controlled through their SUMOylation status [40].

So far, most studies exploring the role of the NPC in genomic regulations focused on protein-coding genes, with few reports indicating its function at other loci (*e*.*g*. RNA polymerase III-transcribed genes [41,42]). In line with the difficulties associated with the analysis of repetitive sequences [43], little is known about the influence of NPCs on the RNA polymerase II (RNAP II)-dependent transcription of transposable elements (TEs). TEs are ubiquitous in eukaryotes and represent a significant fraction of their genomes (reviewed in [44]). The *S*. *cerevisiae* genome hosts five families of LTR-retrotransposons (Ty1 to Ty5) harboring the same basic genomic organization, consisting of two direct long terminal repeats (LTRs) flanking the *GAG/TYA* and *POL/TYB* open reading frames. Like their retrovirus counterparts, Ty retrotransposons replicate by reverse transcription of their RNA genome into a cDNA copy that is stably integrated into the host-cell genome by their self-encoded integrase. Except for Ty3, which is a *Metaviridae* (gypsy-like element), the other yeast Ty elements belong to the *Pseudoviridae* group (copia/Ty1-like elements). Ty1 RNAs account for 0.1–0.8% of total cellular RNA and for up to 10% of mRNAs [45,46], thus representing an important part of the yeast transcriptome that remains mostly unexplored. Ty1 transcription depends on several transcription factors that bind to the Ty1 promoter (*i*.*e*., Gcr1, Ste12, Tec1, Mcm1, Tea1/Ibf1, Rap1, Gcn4, Mot3 and Tye7) or belong to chromatin-remodeling complexes (Swi/Snf, SAGA and ISWI) (reviewed in [47]). In addition, dozens of cellular factors identified as regulators of Ty1 replication still await mechanistic characterization (reviewed in [47]). Among them, subunits of the Nup84 complex were previously scored as necessary for Ty1 retrotransposition in genome-wide screens [48–50], as further validated for the Nup84 subunit [51]. In contrast, a systematic analysis of nucleoporin mutants did not confirm this requirement of the Nup84 complex for Ty1 replication using a distinct reporter system [52]. Finally, this same complex was found to restrict the retrotransposition of an overexpressed Ty3 reporter [51,53]. In view of these conflicting reports, the contribution of the Nup84 complex to retrotransposon biology is far from being understood.

In this study, we investigated the impact of the Nup84 complex on the yeast *S*. *cerevisiae* transcriptome with a special focus on endogenous retrotransposon transcripts. Using a combination of genome-wide approaches and dedicated chromosomal reporter systems, we show that the Nup84 complex represses the transcription of most copies of the Ty1, Ty2 and Ty3 retrotransposons. By genetically disentangling the multiple roles of the Nup84 complex, we further establish that its function in the tethering of the SUMO-protease Ulp1 to the NPC is essential for the transcriptional control of Ty1 retrotransposons. Finally, using a suite of tools to assess each stage of the Ty1 retrotransposition cycle, we demonstrate that, although the increase in Ty1 RNA levels appears minor upon loss-of-function of the Nup84 complex, it has major consequences on Ty1 retrotransposition. The Nup84 complex thus appears essential to keep retrotransposon mobility under control, highlighting the previously underestimated importance of fine-tuning retroelement transcription to limit their propagation in the yeast genome.

## Results

### The Nup84 complex specifically restricts RNA accumulation of LTR-retrotransposons

**Fig 1:**
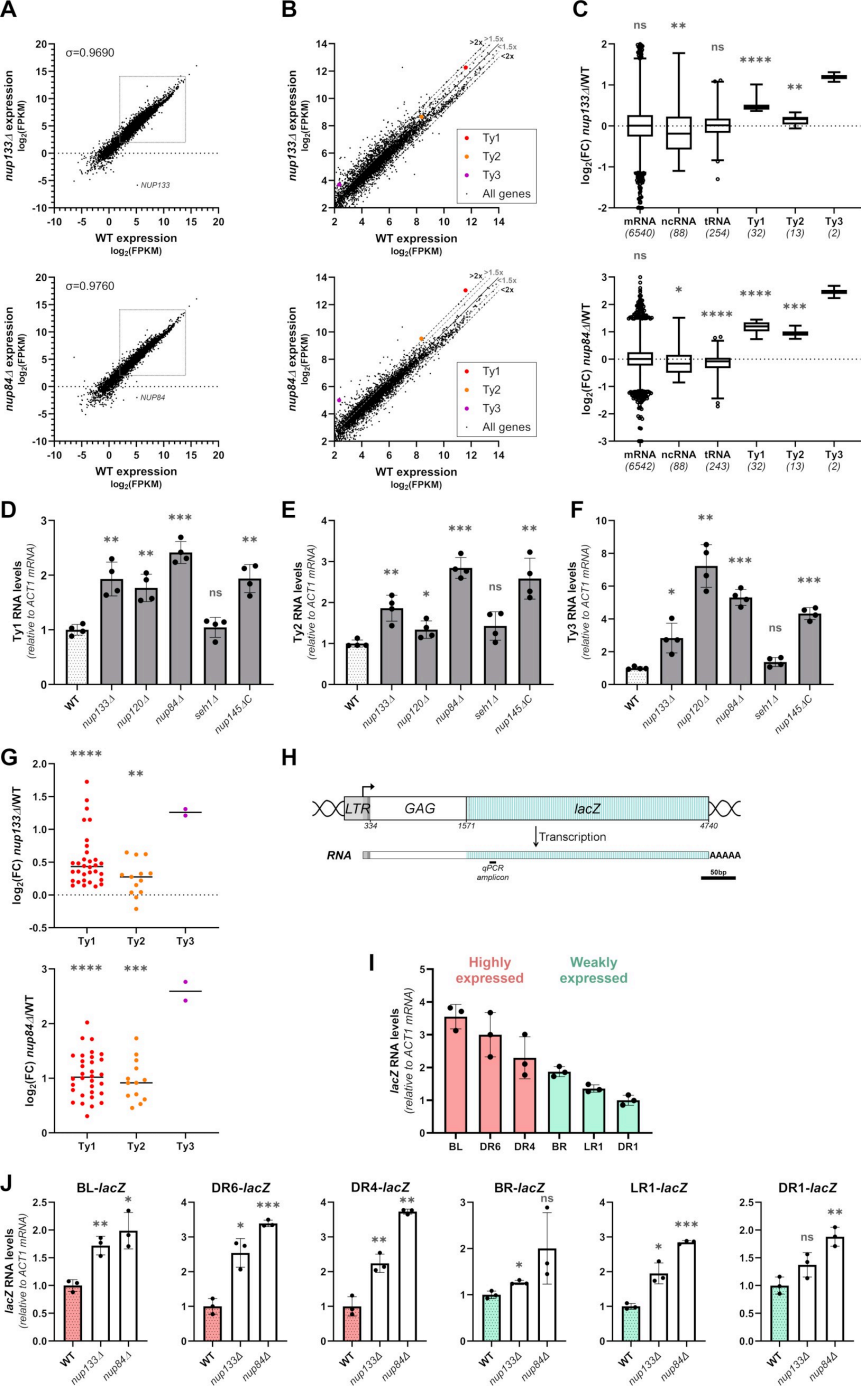
The Nup84 complex specifically restricts Ty RNA accumulation. **(A)** Scatterplots showing RNA abundance in *nup133Δ* (*top panel*) or *nup84Δ* (*bottom panel*) mutants vs WT cells based on RNA sequencing data. RNA levels are represented as log_2_ of fragments per kb per million reads mapped (FPKM). Each value is the average of three biological replicates per strain. The position of *NUP133* and *NUP84* transcripts are highlighted and the Spearman’s correlation coefficient is indicated. The framed area is zoomed in (B). **(B)** Scatterplots as described in (A) but restricted to the range of expression of Ty transcripts. Ty1, Ty2 and Ty3 RNA abundance is highlighted with red, orange and purple dots, respectively. The thresholds of 1.5 or 2-fold change between the 2 strains are indicated by grey or dark dashed lines, respectively. **(C)** Boxplot analysis of log_2_ fold change (FC) of different categories of transcripts in *nup133Δ* (*top panel*) or *nup84Δ* (*bottom panel*) mutants relative to WT cells. The number of transcripts expressed in both strains and considered in each category is indicated in brackets. **(D-F)** Ty1 **(D)**, Ty2 **(E)** and Ty3 **(F)** RNA levels in WT cells and non-essential mutants of the Nup84 complex, as measured by RT-qPCR (mean±SD, n≥3, relative to WT and normalized to *ACT1* mRNAs). **(G)** Analysis of log_2_ fold change (FC) of expression of Ty1, Ty2 and Ty3 copies in *nup133Δ* (*top panel*) or *nup84Δ* (*bottom panel*) mutants relative to WT cells. Only reads assigned to a single locus in the genome were considered for this analysis. **(H)** Scheme of the Ty1-*lacZ* reporters. The *lacZ* gene is fused in-frame to *GAG* at individual endogenous Ty1 copies downstream of their cognate promoter sequences (at coordinate 1571 of Ty1-H3) [59]. The position of the qPCR amplicon used in (I) and (J) is indicated. Scale bar, 50bp. **(I)** Ty1-*lacZ* fusion RNA levels in WT cells, as measured by RT-qPCR (mean±SD, n = 3, relative to DR1 and normalized to *ACT1* mRNAs). The Ty1 copies are ranked according to their RNA levels and annotated as highly or weakly expressed in [59]. **(J)** Ty1-*lacZ* RNA levels of different Ty1-*lacZ* fusions, as measured by RT-qPCR in WT cells and *nup133Δ* or *nup84Δ* mutants (mean±SD, n = 3, relative to WT and normalized to *ACT1* mRNAs). ns, not significant; * p<0.05; ** p<0.01; *** p<0.001; **** p<0.0001, two-sided Wilcoxon rank-sum test (panels C,G) or Welch’s t test with comparison to the WT strain (D,E,F,J). Note that the low number of Ty3 loci precludes any statistical analysis (C,G).

To investigate the role of the Nup84 complex on gene expression, we performed a transcriptomic analysis in two different mutants deleted for non-essential subunits of this NPC subcomplex (*nup133Δ* and *nup84Δ*). To extend our analysis beyond protein-coding genes and include retrotransposon transcripts, which are mostly deadenylated [54], stranded RNA-seq was performed after depletion of the highly-abundant ribosomal RNAs but without selection for polyadenylated RNAs. Since Ty1 retrotransposition is optimal at 20–22°C [55] and most Nup84 complex mutants are thermosensitive (S1A Fig), all the experiments were further conducted at 25°C to guarantee both Ty1 mobility and the growth of mutant strains. Three independent cell cultures were analyzed for each genotype with a high level of reproducibility between samples of the same strain (S1B Fig). Globally, the mean transcriptomes of *nup133Δ* and *nup84Δ* cells barely differed from that of WT cells (Fig 1A). In these strains, 32 full-length copies of Ty1, 13 full-length copies of Ty2 and 2 full-length copies of Ty3 produce RNAs suitable for reverse transcription while the 3 copies of Ty4 only yield truncated transcripts lacking essential sequences for reverse transcription, and the only Ty5 copy is truncated and not expressed [56,57]. Since the sequences of the Ty copies within each family are very close, Ty RNAs are usually discarded from genomic analyses that focus on single-mapping reads. To circumvent this issue, we used multi-mapping reads, randomly assigned to these repeated sequences of the genome, in order to evaluate the bulk of Ty RNAs in WT and *nup* transcriptomes. In this way, we confirmed that Ty1 transcripts are among the most abundant RNAs in WT cells while Ty2 and Ty3 transcripts are less abundant, correlating with lower genomic copy number [57] (Fig 1B). Most importantly, our analysis revealed that Ty1, Ty2 and Ty3 transcripts are over-represented about 1.5-fold and 2-fold in the transcriptome of *nup133Δ* and *nup84Δ* mutants, respectively (Fig 1B). Differential expression analysis further confirmed a consistent increase in Ty1 and Ty3 RNA levels in both Nup84 complex mutants (Fig 1C), while Ty2 transcripts were mostly up-regulated in *nup84Δ* cells. Notably, these tendencies were not observed for mRNAs, tRNAs or other ncRNAs (Fig 1C), demonstrating that Nup133 and Nup84 specifically target LTR-retrotransposon transcripts.

To determine whether this unreported function of Nup133 and Nup84 is shared by the other components of the Nup84 complex, we measured Ty1, Ty2 and Ty3 RNA levels by RT-qPCR in deletion mutants for all non-essential Nups of the Nup84 complex in the S288C genetic background. As a control for the ability of our RT-qPCR measurement to distinguish Ty families, deletion of *SPT3*, encoding a SAGA subunit specifically required for Ty1 transcription, strongly reduced Ty1 RNA levels while it did not affect Ty3 expression (S1C Fig). Our assay further scored a significant ~2-fold increase in Ty1 RNA levels in four of the five tested Nup84 complex mutants (Fig 1D). Similarly, Ty2 and Ty3 RNA levels increased in all the mutants except *seh1Δ* (Fig 1E and 1F), in line with the less severe phenotypes displayed by this deletant as compared to other Nup84 complex mutants [58], possibly due to the peripheral position of the Seh1 protein in the structure of the complex [29]. Altogether, these data highlight an unexpected function of the Nup84 complex in limiting the accumulation of RNAs from LTR-retrotransposons.

Dozens of Ty copies are scattered throughout the yeast genome and could be differently affected by NPCs. To address whether the Nup84 complex specifically targets certain Ty within the genome or globally regulates the expression of all the LTR-retrotransposon copies, we sought to discriminate the expression changes of the distinct genomic Ty loci (Fig 1G). For this purpose, we restricted our differential expression analysis to single-mapping reads encompassing copy-specific sequence variations, *e*.*g*. SNPs. The analysis based on these reads, which represent about 20%, 20% and 70% of Ty1, Ty2 and Ty3 mappers, respectively, gave fully consistent results with the multi-mapping read analysis, with similar median log_2_ fold changes (FC) for Ty1 copies (single-mapping: *nup133Δ*: 0.43, *nup84Δ*: 1.02; multi-mapping: *nup133Δ*: 0.46 and *nup84Δ*: 1.2; compare Fig 1G and 1C). Moreover, the single-mapping analysis showed that all the 32 genomic copies of Ty1 were up-regulated in both *nup133Δ* and *nup84Δ* mutants, although to different extent depending on the copies (Fig 1G). Similarly, the expression of the two Ty3 copies was highly up-regulated in both mutants of the Nup84 complex. In contrast, Ty2 copies were all up-regulated in the *nup84Δ* mutant while some of them were not affected in the *nup133Δ* mutant, explaining the lower overall impact of the *nup133Δ* mutant compared to the *nup84Δ* mutant on Ty2 transcript levels (Fig 1C).

The estimation of the transcript levels derived from different TE genomic copies remains partially biased with such transcriptomic analyses. In particular, single-mapping will retain more reads on older sequences that have accumulated more SNPs than younger elements [43]. To precisely evaluate the global impact of the Nup84 complex on distinct copies of Ty1, we used integrated Ty1-*lacZ* fusions previously designed to study the transcription of individual Ty1 copies at their genomic location [59]. In these constructions, the *lacZ* gene was fused at position 1571 of each Ty1 copy to keep an intact Ty1 promoter that extends over 1kb from the 5’LTR to a large part of the *GAG* ORF (Fig 1H). RT-qPCR measurement of the RNA levels produced from representative Ty1-*lacZ* copies revealed a range of expression levels in WT cells, in agreement with earlier β-galactosidase activity measurements [59] (Fig 1I). Importantly, Ty1-*lacZ* RNA levels increased for every Ty1 copy tested upon deletion of *NUP133* and *NUP84* (Fig 1J), similarly to the overall accumulation of RNAs produced by all the endogenous Ty1 copies of these strains (S1D Fig), and in agreement with our single-read mapping analysis. While slight variations in the up-regulation levels were noticeable between Ty1-*lacZ* fusions or Ty1 copies, they did not correlate with the basal expression of the Ty1 copy in WT cells (Figs 1I and S1F), its position on the Watson or Crick strand or on the chromosome arm, or the close proximity of a *tDNA* gene (S1E and S1F Fig). Taken together, these results demonstrate that the Nup84 complex broadly restricts RNA accumulation from Ty LTR-retrotransposons all over the genome.

### The Nup84 complex regulates the transcription of Ty LTR-retrotransposons

**Fig 2:**
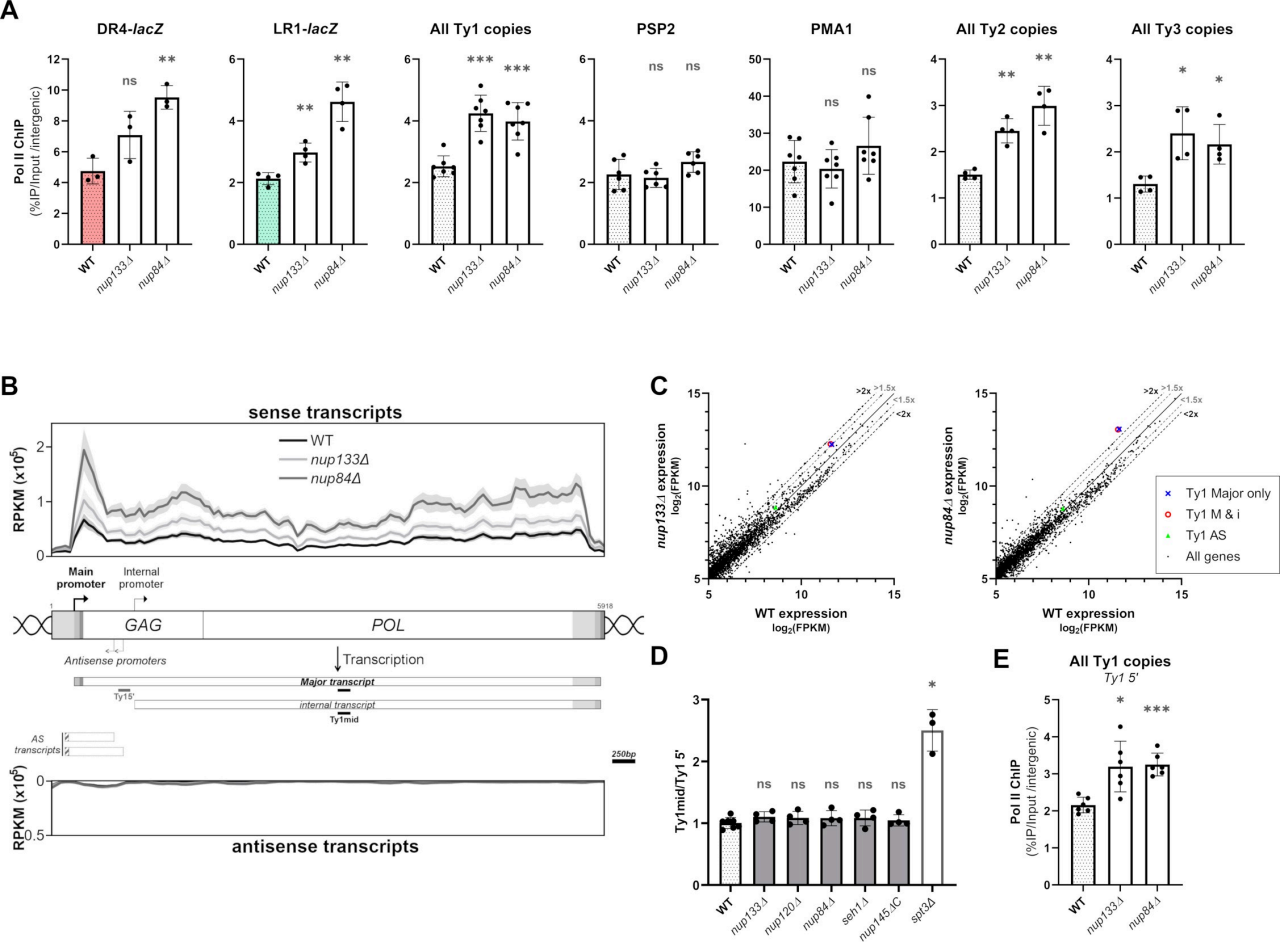
The Nup84 complex regulates the expression of LTR-retrotransposons at the transcriptional level. **(A)** RNAP II occupancy on the DR4 and LR1 Ty1-*lacZ* fusions, all Ty1 genomic copies, *PMA1* or *PSP2* loci, and all Ty2 and Ty3 genomic copies, as determined by ChIP in WT, *nup133Δ* and *nup84Δ* cells. Values (mean±SD, n≥3) are expressed as a percentage of IP and normalized to an intergenic region. **(B)** *Upper and lower panels*: Metagene analysis showing Ty1 RNA-seq sense and antisense read counts in WT, *nup133Δ* and *nup84Δ* cells, averaged over all 32 genomic Ty1 elements and represented as reads per kb per million (RPKM; mean±standard error, n = 3). *Mid panel*: Ty1 locus organization and expression. Long Terminal Repeats (LTR) flank two overlapping ORFs (*GAG* and *POL*). The 5.7 kb Ty1 major transcript starts at the U3/R junction of the 5′ LTR and ends at the R/U5 junction of the 3′ LTR (U3, R and U5 regions are indicated from light grey to dark grey). The 5 kb internal Ty1 RNA species is transcribed from an internal promoter located around 800bp downstream of the main promoter. Major antisense (AS) noncoding RNAs starting from two distinct promoters are depicted, with their reported heterogenous 3’ ends represented as shaded boxes (according to [109,110]). The position of the qPCR amplicons used in (D) are shown, the Ty1mid amplicon being used for RT-qPCR measurement of Ty1 RNA levels in all the figures except Fig 2D and 2E. Scale bar, 250bp. **(C)** Scatterplots showing expression levels of cellular RNAs in *nup133Δ* (*left*) or *nup84Δ* (*right*) mutants vs WT strains as described in Fig 1B. Expression of Ty1 based on reads either uniquely assigned to major transcripts (*blue cross*) or assigned to both major and internal transcripts (*red circle*) is specified. The abundance of antisense Ty1 RNAs is also highlighted (*green triangle*). **(D)** Ty1 RNA levels in WT cells and non-essential mutants of the Nup84 complex, as measured by RT-qPCR using Ty1 5’ and Ty1mid amplicons, which hybridize only with major transcripts and all sense transcripts, respectively. The ratio of values obtained with Ty1mid and Ty1 5’ is represented (mean±SD, n≥3). The *spt3Δ* mutant is used as a positive control for induction of the internal promoter and specific increase of internal transcripts. **(E)** RNAP II occupancy at the 5’ end of Ty1 genomic copies determined by ChIP using the Ty1 5’ amplicon in WT, *nup133Δ* and *nup84Δ* cells. Values (mean±SD, n≥3) are expressed as a percentage of IP and normalized to an intergenic region. ns, not significant; * p<0.05; ** p<0.01; *** p<0.001, Welch’s t test with comparison to the WT cells.

An increase in RNA levels could stem from changes in transcription or RNA stability. Since Ty1 RNAs have been described as particularly stable [60] and considering that mutations of the Nup84 complex have already been associated with changes in transcription of reporter genes [35,36], we analyzed the influence of the Nup84 complex on Ty1 transcription. For this purpose, we used Ty1-*lacZ* fusions to analyze the recruitment of RNAP II on individual Ty1 copies by ChIP-qPCR (Fig 2A). In WT cells, RNAP II occupancy was higher at the *DR4-lacZ* locus than at the *LR1-lacZ* locus, in agreement with their status of highly- and weakly-expressed Ty1 copies, respectively (Figs 2A and 1I) [59]. Upon deletion of either *NUP133* or *NUP84*, RNAP II levels increased at both loci and on all genomic Ty1 elements with respect to distinct intergenic sequences (Figs 2A and S2A–2C). Importantly, in the same mutants, RNAP II occupancy was unchanged at a highly-transcribed control gene (*PMA1*, Fig 2A), or at a locus as weakly transcribed as Ty1 loci (*PSP2*, Fig 2A). Furthermore, while Ty2 and Ty3 copies were undetectably recruiting RNAP II in WT cells, significant RNAP II ChIP signals were scored at these loci upon deletion of the subunits of the Nup84 complex (Fig 2A). This set of results demonstrates that the Nup84 complex globally impacts RNAP II occupancy at Ty1, Ty2 and Ty3 elements. While Ty1 and Ty2 are closely related LTR-retrotransposons, Ty3 belongs to another superfamily, suggesting that the Nup84 complex keeps transcription of evolutionarily distant LTR-retrotransposons at low levels.

Different promoters have been identified in Ty1, yielding sense and antisense Ty1 RNA species (Fig 2B) (reviewed in [47]). While only the major transcript contains all the sequences required for reverse transcription, internal and antisense transcripts have been assigned roles in the regulation of retrotransposition. In particular, the internal transcript encodes p22 and p18, which are truncated Gag proteins involved in the copy number control of Ty1 mobility [61]. Although internal transcripts are weakly expressed in WT cells, this internal promoter is specifically induced in certain situations (*e*.*g*. *spt3* mutants [61]). To determine precisely which of the promoter(s) located within Ty1-*lacZ* fusions are repressed by the Nup84 complex, we first performed a strand-specific metagene analysis of Ty1 RNA-seq reads, which revealed that Ty1 RNA accumulation is observed all over Ty1 sense transcripts in *nup133Δ* and *nup84Δ* cells, with the same prominent peaks at the main and internal promoters as in WT cells (Fig 2B). To complement this observation, we further re-analyzed our RNA-seq data by separately quantifying reads assigned to three different Ty1 regions, as previously described [62]: the 5’ part of the coding strand specific to the major transcript (“Ty1 Major only”; Fig 2C), the 3’ part of the coding strand shared by both the major and the internal transcripts (“Ty1 M & i”, Fig 2C), and the 3’ part of the non-coding strand corresponding to antisense RNAs (“Ty1 AS”, Fig 2C). Read counting indicated that antisense RNA levels remained unchanged in the mutants compared to WT cells, whereas both “Ty1 Major only” and “Ty1 M & i” transcripts were equally abundant in WT cells and similarly over-represented in *nup133Δ* and *nup84Δ* mutants (Fig 2C). In addition, RT-qPCR analysis comparing the amount of RNAs measured by two different qPCR amplicons in the “Ty1 Major only” or “Ty1 M & i” regions indicated that unlike in the control *spt3Δ* mutant, the major and the internal promoters are not differentially induced in any of the Nup84 complex mutants (Fig 2D). To independently quantify the derepression of internal transcription in a more sensitive assay, we probed *nup* mutants for the presence of p22 and p18, which both specifically arise from translation of Ty1i RNA species [61]. This analysis revealed that p18, but not p22, became detectable in *nup* mutant cells, supporting that alike major transcripts, internal transcripts are also induced in the absence of the Nup84 complex (S2D Fig). In agreement with our RNA-seq and RT-qPCR analyses, the ratios of p22/p18 over the specific translation products of the major transcripts (*i*.*e*. p49/p45-Gag) were unchanged in *nup* mutants (S2E Fig), supporting that both the main and the internal promoters are similarly derepressed in the absence of the Nup84 complex. Of note, the increased recruitment of RNAP II at the 5’ end of Ty1 upon deletion of *NUP133* and *NUP84* (Fig 2E) strongly suggests that the Nup84 complex restricts the transcription of Ty1 at the initiation level.

Altogether, these results demonstrate that the Nup84 complex represses the transcriptional activity of Ty1, Ty2 and Ty3 LTR-retrotransposons, thereby limiting the quantity of transcripts that serve as templates for both translation and reverse transcription.

### The Nup84 complex represses Ty1 expression through Ulp1-dependent SUMOylation processes

**Fig 3:**
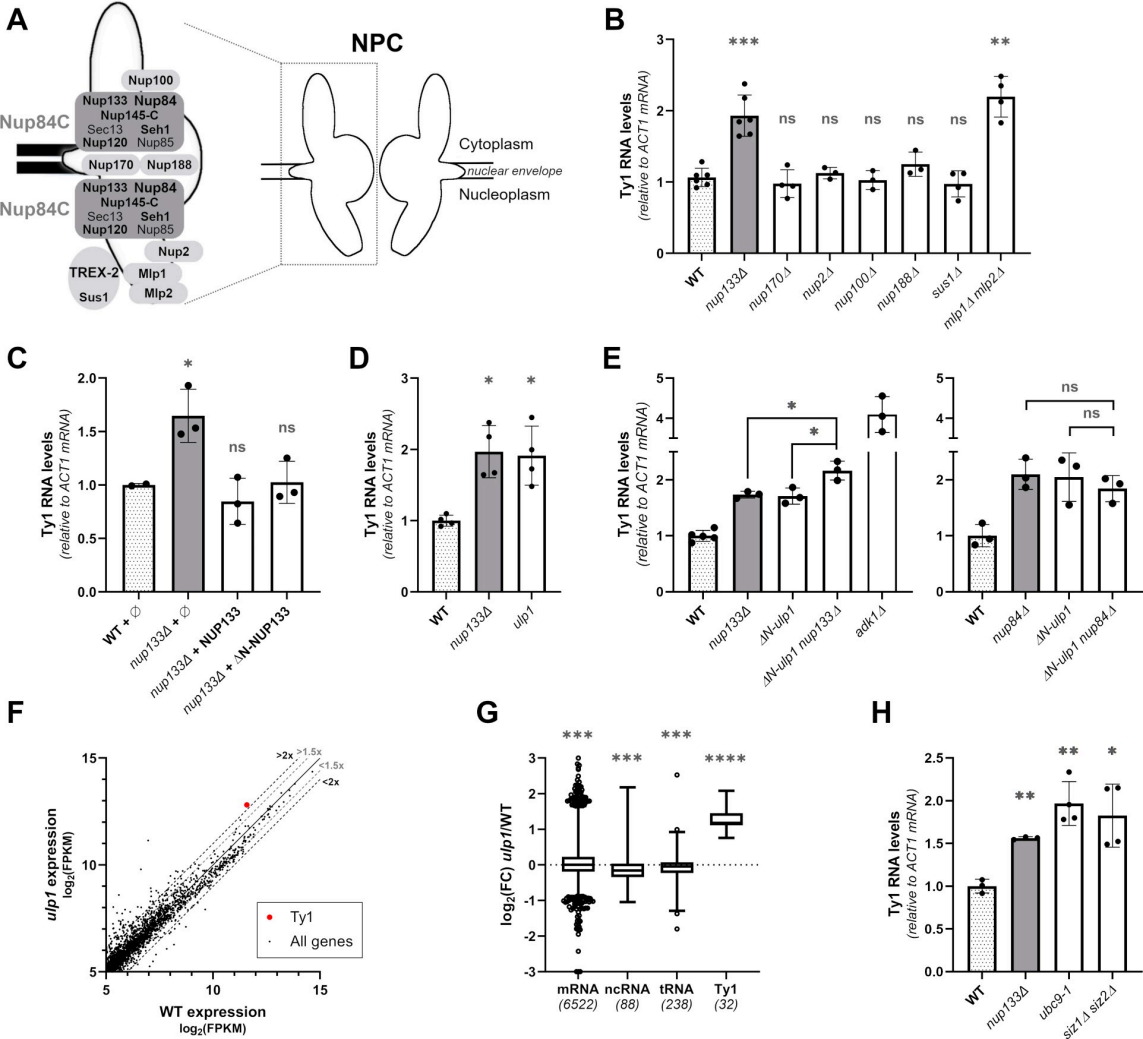
The Nup84 complex represses Ty1 expression through the tethering of the SUMO protease Ulp1 at the NPC. **(A)** Schematic representation of the structural organization of the NPC, highlighting the relative position of the nucleoporins analyzed in this study. Nup84 complex (Nup84C) subunits and other nucleoporins mutated here appear in bold. **(B)** Ty1 RNA levels in WT cells and mutants of nucleoporins and NPC-associated proteins, as measured by RT-qPCR (mean±SD, n≥3, relative to WT and normalized to *ACT1* mRNAs). Ty1 RNA levels in the *nup133Δ* mutant are shown as a control of Ty1 induction. **(C)** Ty1 RNA levels in *nup133Δ* cells carrying either the pUN100 (ø), the pUN100-protA-NUP133 or the pUN100-protA-*Δ*N-NUP133 plasmids, as measured by RT-qPCR (mean±SD, n≥2, relative to WT and normalized to *ACT1* mRNA values). **(D)** Ty1 RNA levels in WT cells and the *ulp1* mutant, as measured by RT-qPCR (mean±SD, n≥3, relative to WT and normalized to *ACT1* mRNAs). Ty1 RNA levels in the *nup133Δ* mutant are shown as a control of Ty1 induction (same values as panel B). **(E)** Ty1 RNA levels in WT cells and single or double *nup133Δ ΔN-ulp1* or *nup84Δ ΔN-ulp1* mutants, as measured by RT-qPCR (mean±SD, n≥3, relative to WT and normalized to *ACT1* mRNA). The *adk1Δ* mutant was used as a characterized Ty1 RNA-accumulating control mutant, in which up-regulation of the Tye7 transcription factor leads to enhanced Ty1 expression [72]. **(F)** Scatterplot showing expression levels of cellular RNAs in *ulp1* mutants vs WT cells as described in Fig 1B. The abundance of total Ty1 RNAs is highlighted with a red dot. The thresholds of 1.5 or 2-fold change between the 2 strains are indicated by a grey or dark dashed line, respectively. **(G)** Boxplot analysis of log_2_ fold change (FC) of different categories of transcripts in *ulp1* mutant relative to WT cells. The number of transcripts expressed in both strains and considered in each category is indicated in brackets. Only reads assigned to a single locus in the genome were considered for this analysis. **(H)** Ty1 RNA levels in WT cells and *ubc9-1* or *siz1Δ siz2Δ* mutants, as measured by RT-qPCR (mean±SD, n≥3, relative to WT and normalized to *ACT1* mRNAs). ns, not significant; * p<0.05; ** p<0.01; *** p<0.001, **** p<0.0001, Welch’s t test with comparison to WT (B,C,D,H) or as indicated (E), or two-sided Wilcoxon rank-sum test (G).

We next asked by which mechanisms the Nup84 complex could repress Ty transcription. Although this regulation occurs for different families of retrotransposons, we focused our study on Ty1 transcripts, which are far more abundant than Ty2 and Ty3 RNAs in WT cells (about 10-fold and 600-fold, respectively; Fig 1B). We first investigated whether the derepression of Ty1 transcription was specific of mutants of the Nup84 complex, or could be generally observed when NPC structure or nucleocytoplasmic transport are compromised. For this purpose, we systematically assayed Ty1 expression by RT-qPCR in representative nucleoporin mutants with characterized defects in the NPC permeability barrier (*nup170Δ*, *nup188Δ*, [63]), NLS-dependent protein import (*nup2Δ*, [64]) or tRNA export (*nup100Δ*, [65]) (Fig 3A). None of these mutants displayed altered Ty1 RNA levels as compared to WT, in contrast with the *nup133Δ* mutant (Fig 3B), supporting the fact that Ty1 derepression is not the mere consequence of impaired nucleocytoplasmic transport in Nup84 complex mutants.

We then examined if one of the previously described roles of the Nup84 complex in mRNA export and NPC distribution could underlie its impact on Ty1 transcription [29]. Although alterations in mRNA export can impact transcription [66], independently impairing mRNA export through the inactivation of the NPC-associated mRNA export complex TREX-2 (*sus1*Δ) did not impact Ty1 expression levels (Fig 3B). Likewise, expression of a separation-of-function *nup133* allele, which solely exhibits the NPC clustering phenotype *(ΔN-nup133*; [67]), fully complemented Ty1 RNA expression defects (Fig 3C). Altogether, our data indicates that neither the mRNA export defects nor the NPC clustering observed in Nup84 complex mutants are the cause of Ty1 derepression.

Strikingly, the increase in Ty1 RNA levels observed in Nup84 complex mutants was fully phenocopied by the simultaneous inactivation of both nuclear basket proteins Mlp1 and Mlp2 (*mlp1Δ mlp2Δ*, Fig 3B). Components of the Nup84 complex and of the nuclear basket (*e*.*g*. Mlp1, Mlp2, Nup60) have in common to contribute to the tethering of the SUMO-deconjugating enzyme Ulp1 to NPCs [31,68,69], although the direct interacting partners and the molecular bases for this shared function are still unknown. For this reason, we directly assayed whether *ULP1* inactivation could similarly impact Ty1 expression. RT-qPCR analyses revealed increased Ty1 RNA levels in a *ulp1* thermosensitive mutant impacting both Ulp1 NPC localization and catalytic activity (*ulp1-333*, referred to as *ulp1*; [70]) (Fig 3D), as well as in a *ulp1* truncation mutant lacking its pore targeting domain (*ΔN-ulp1*, [71]; Fig 3E). Further transcriptomic analysis of *ulp1* cells confirmed a specific increase of Ty1 RNAs, with other transcript categories, in particular RNAP II-transcribed mRNAs, being globally unaffected, as in Nup84 complex mutant cells (Fig 3F and 3G). Among the few protein-coding genes that were nonetheless commonly up- or down-regulated in *nup133Δ*, *nup84Δ* and *ulp1* mutants (FC>2, S1 Table), none of them was previously reported as either a Ty1 Retromobility Host Factor (RHF) or a Restrictor of Ty Transposition (RTT) [47] (S3A Fig). Still, a subset of these shared targets (n = 9; S3A Fig) are involved in gene expression *largo sensu* and could represent yet uncharacterized Ty1 RNA regulators, indirectly accounting for the observed Ty1 derepression scored in *nup* or *ulp1* mutants. However, their inactivation did not result in changes in Ty1 RNA levels (S3B Fig). Similarly, loss of the Seh1 subunit of the Nup84 complex neither triggers the changes in SUMOylation patterns typically caused by the loss of Ulp1 tethering in other Nup84 complex mutants (S3C Fig), nor impacts Ty1 expression (Fig 1D). Altogether, these observations support that the Nup84 complex and Ulp1 do not interfere with the expression of Ty regulators but rather impact their SUMOylation status.

To further confirm that the Ty1 phenotype of Nup84 complex mutants originates from defective Ulp1 tethering and/or activity, we performed epistasis experiments by combining *NUP133* or *NUP84* deletions with the *ΔN-ulp1* truncation. The *nup133Δ ΔN-ulp1* double mutant did not display a strong synergistic effect as compared to the single mutants (Fig 3E, *left panel*), although our assay was not saturated as revealed by the higher Ty1 RNA accumulation detected in the *adk1Δ* mutant [72]. Furthermore, the *nup84Δ ΔN-ulp1* double mutant phenotype was similar to the ones scored in the respective single mutants (Fig 3E, *right panel*). Altogether, our data establish that the Nup84 complex regulates Ty1 transcription through the tethering of the SUMO-deconjugating enzyme Ulp1.

Importantly, Ulp1 acts at distinct stages in the SUMOylation process, both activating SUMO precursors, an absolute prerequisite for SUMO conjugation, and removing SUMO moieties from certain targets. In addition, anchoring of Ulp1 at the NPC was proposed to prevent unscheduled deSUMOylation in the nucleoplasm [31,68,71]. To define whether the Ty1 derepression observed in *ulp1* mutants is caused by decreased SUMOylation or impaired deSUMOylation, we assayed Ty1 expression in other mutants of the SUMOylation machinery (Fig 3H). Inactivation of the unique SUMO-conjugating enzyme, Ubc9, or the simultaneous deletion of the two major SUMO ligases, Siz1 and Siz2, triggered a significant increase in Ty1 RNA levels, comparable to the one observed in Nup84 complex or *ulp1* mutants. These data, together with the limited impact of Nup84 complex mutants on cellular SUMOylation patterns [31,36] (S3C Fig), support the fact that Ty1 activation originates from decreased SUMOylation of some cellular factors commonly affected in these different backgrounds. Taken together, our results support a model in which the Nup84 complex limits Ty1 transcription by regulating SUMOylation processes through the tethering of the SUMO-deconjugating enzyme Ulp1 at the NPC.

### The Nup84 complex-dependent control of Ty1 transcription prevents unrestrained retrotransposition

**Fig 4:**
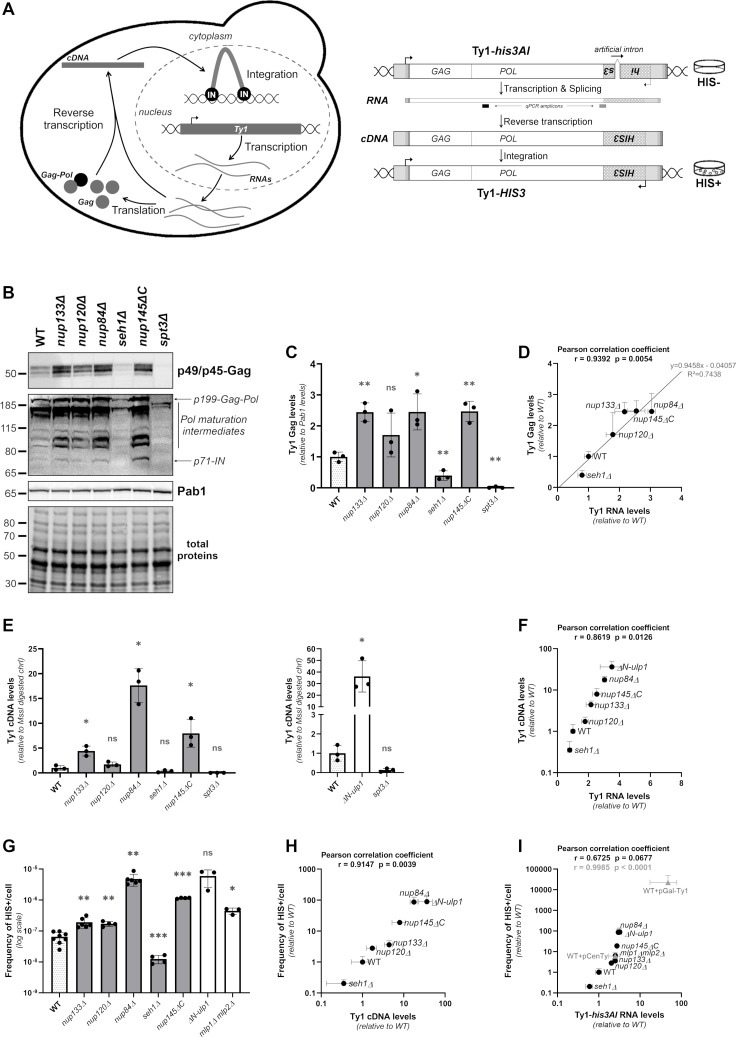
The Nup84 complex prevents unrestrained retrotransposition. **(A)** *Left panel*: The Ty1 replication cycle. A Ty1 element is first transcribed into RNAs that are exported to the cytoplasm. Ty1 RNAs are subsequently translated into Gag and Gag-Pol proteins and reverse transcribed into cDNAs by the reverse transcriptase, which is derived from the Gag-Pol polyprotein maturation into Gag, protease, integrase (IN) and reverse transcriptase proteins. IN binds the Ty1 cDNA, which is imported into the nucleus and integrated into the yeast genome. *Right panel*: Principle of the retrotransposition assay. The *his3AI* retrotransposition reporter gene consists of a *HIS3* gene inserted in the opposite orientation in the 3’ untranslated region of Ty1 and interrupted by an artificial intron, in a spliceable orientation in the Ty1 transcript. Upon splicing and reverse transcription, a Ty1-*HIS3* cDNA is produced which confers a His+ phenotype to the cells when integrated into the host genome. qPCR amplicons used for total Ty1 or specific Ty1-*his3AI* transcript quantification are indicated in black and grey, respectively. **(B)** Whole cell extracts of the indicated strains analyzed by western blotting using anti-VLP antibodies, revealing p49/p45-Gag proteins, and anti-integrase antibodies, revealing all Pol maturation intermediates leading to integrase (p71-IN). Pab1 and total proteins are used as a loading control. Molecular weights are indicated (kDa). **(C)** Quantification of Gag levels from (B) (mean±SD, n = 3, relative to WT and normalized to Pab1 protein levels). **(D)** Total Gag protein levels (mean±SD, n = 3, relative to WT, values from Fig 4C) are plotted as a function of total Ty1 RNA levels (mean±SD, n≥3, relative to WT, values from S4B Fig). The Pearson correlation coefficient and associated p-value are indicated. The equation and R^2^ coefficient obtained from the linear regression are also indicated. **(E)** Total Ty1 cDNA levels in WT cells and non-essential mutants of the Nup84 complex (*left panel*) or Ulp1 (*right panel*), as measured by qPCR (mean±SD, n = 3, relative to WT and normalized to the values of a chromosome I genomic locus digested by MssI). The *spt3Δ* mutant is used as a control for qPCR specificity because Ty1 expression is strongly decreased in this mutant. **(F)** Total Ty1 cDNA levels (mean±SD, n = 3, relative to WT, values from Fig 4E) are plotted as a function of total Ty1 RNA levels (mean±SD, n≥3, relative to WT, values from S4B Fig) in the indicated strains. The Pearson correlation coefficient and associated p-value are indicated. **(G)** Retrotransposition frequencies (log scale, mean±SD, n≥3) of Ty1-*his3AI* reporter in WT cells and non-essential mutants of the Nup84 complex, *ΔN-ulp1* or *mlp1Δ mlp2Δ* mutant. **(H)** Retrotransposition frequencies (mean±SD, n≥3, relative to WT, data from Fig 4G) are plotted as a function of total Ty1 cDNA levels (mean±SD, n = 3, relative to WT, values from Fig 4E) in the indicated strains. The Pearson correlation coefficient and associated p-value are indicated. **(I)** Retrotransposition frequencies (mean±SD, n≥3, relative to WT, data from Fig 4G) are plotted as a function of Ty1-*his3AI* RNA levels (mean±SD, n≥3, relative to WT, values from S4G Fig) in the indicated strains. The Pearson correlation coefficient and associated p-value are indicated in black. Retrotransposition frequencies and Ty1-*his3AI* RNA levels in WT cells expressing a Ty1-*his3AI* reporter from pCenTy (centromeric) or pGAL-Ty1 (multicopy) plasmids are represented in grey. The Pearson correlation coefficient and associated p-value calculated upon inclusion of the plasmid values are indicated in grey. Note that all the strains used in this figure carry a chromosomal Ty1-*his3AI* reporter. ns, not significant; * p<0.05; ** p<0.01, *** p<0.001, Welch’s t test with comparison to the WT strain.

RNAs are pivotal in the retrotransposition process as they serve as templates for both the translation of structural and enzymatic proteins and for the reverse transcription into cDNAs (Fig 4A, *left panel*). To explore the consequences of the increase of Ty1 RNA levels observed in Nup84 complex mutants on Ty1 retrotransposition, we quantified the efficiency of each stage of the retrotransposition cycle in WT, Nup84 complex and *ulp1* mutant cells.

First, to assess the export and the translation of Ty1 RNAs, we evaluated the levels of Ty1-encoded proteins by western blot analysis. Increased Gag and integrase (IN) protein levels were detected in all Nup84 complex mutants except *seh1Δ* (Fig 4B and 4C), as well as in *ulp1* mutant cells (S4A Fig), mirroring Ty1 RNA accumulation (S4B Fig), and further confirming that Ty1 RNAs are normally exported to the cytoplasm. The remarkable linear correlation between RNA and Gag variations (Fig 4D; Pearson correlation coefficient r = 0.9392; α~1) further indicates that the increase in Ty1 transcription in Nup84 complex mutants equivalently impacts Gag protein levels.

Next, we examined the reverse transcription of Ty1 mRNAs in the same mutants. To accurately monitor Ty1 cDNA variations, we adapted a strategy previously developed to quantify cDNAs from retrotransposons in plants [73–75] (see Material and Methods). In this assay, adapters are ligated to non-integrated Ty1 cDNA molecules (that unlike genomic Ty1 copies, display free ends), allowing specific qPCR-based quantification (S4C Fig). As anticipated, no cDNA molecules were detected in the *spt3Δ* mutant (Fig 4E) [76] or in the absence of adapters (S4D Fig), validating that our assay does not detect Ty1 genomic copies. In addition, we quantified about 0.1 molecule of Ty1 cDNA per WT haploid genome (S4E Fig), a value in accordance with previous reports [77,78]. Given the abundance of Ty1 RNAs in the cellular transcriptome (Fig 1B), this result confirms the low efficiency of the post-translational steps of Ty1 replication [79]. Finally, our measured values fell within the range of previously reported quantifications obtained from Southern-based experiments for characterized cDNA-accumulating mutants (*i*.*e*. *fus3Δ*, *rrm3Δ;* [78,80,81]; S4F Fig). When this approach was applied to Nup84 complex and *ulp1* mutant cells, a sharp increase in Ty1 cDNA molecules was scored as a result of Ty1 RNA accumulation, suggesting that Ty1 transcript levels may be limiting for reverse transcription (Fig 4E). The accumulation of Ty1 RNAs therefore leads to an exponential increase in cDNA molecules through reverse transcription in Nup84 complex and *ulp1* mutants (Fig 4F), as exemplified by the *nup84Δ* mutant, in which a 3-fold increase in RNA levels results in an 18-fold increase in cDNA molecules.

To finally determine the consequences of increased Ty1 RNA levels on Ty1 retrotransposition frequency in NPC mutants, we used a chromosomal Ty1-*his3AI* reporter derived from a natural retrotransposition event [82] and conferring histidine prototrophy to cells having undergone a novel retrotransposon insertion (Fig 4A, *right panel*) [83]. Ty1-*his3AI* RNA levels were increased in all *nup* and *ulp1* mutants as compared to WT cells (except *seh1Δ*; S4G Fig), confirming that the Ty1-*his3AI* reporter is targeted by the Nup84 complex as all the endogenous Ty1 copies (S4B Fig). Consistent with previous quantifications of Ty1 RNA, protein and cDNA levels, the retrotransposition frequency significantly increased in all Nup84 complex, *mlp1Δ mlp2Δ* and *ulp1* mutants, except *seh1Δ* (Fig 4G). This observation supports the fact that cDNA molecules can readily enter the nucleus in these *nup* mutants, in line with their reported lack of defects in the classical nuclear import pathway used by the Ty1 integrase [31,84]. As expected, cDNA levels and retrotransposition frequencies were strongly correlated in the different mutants (Pearson correlation coefficient r = 0.9147) (Fig 4H). However, similarly to the amplification scored above at the reverse transcription stage, variations in cDNA levels markedly resulted in even more important differences in retrotransposition frequencies in Nup84 complex and *ulp1* mutant cells. For example, in the *nup84Δ* mutant, the 18-fold increase in cDNA molecules triggered by a 3-fold RNA accumulation led to an 86-fold increase in the frequency of retrotransposition. In *ΔN-ulp1* cells, a 36-fold increase in cDNA amounts caused by a 3.5-fold increase in Ty1 RNAs results in an 89-fold increase in retrotransposition. Although a strong correlation between RNA levels and retrotransposition frequencies was still observed in all the strains (Pearson correlation coefficient r = 0.6725) (Fig 4I), our results establish that the Ty1 RNA accumulation triggered by Nup84 complex deficiencies is amplified at both the reverse transcription and the retrotransposition stages.

Our data could reflect the fact that the Nup84 complex and Ulp1 also act on the Ty1 cycle in a post-transcriptional manner, or that any increase in Ty1 mRNA levels would be similarly amplified at the retrotransposition stage. To distinguish between both possibilities, we specifically interfered with Ty1 RNA levels in WT cells by using Ty1-*his3AI* reporters carried either by a centromeric, low copy plasmid, under the control of its own promoter (pCenTy), or by a multicopy plasmid, under the control of the strong inducible *GAL1* promoter (pGAL-Ty1). Strikingly, the retrotransposition frequencies of these reporters were correlated with the differences in their Ty1-*his3AI* RNAs levels, and aligned on the correlation established for Nup84 complex and *ulp1* mutants (Pearson correlation coefficient r = 0.9985) (Fig 4I). This contrasts with described mutants with proposed post-transcriptional alterations of Ty1 dynamics (*e*.*g*. ribosome mutants [85]), in which Ty1 RNAs levels and retrotransposition rates are not correlated. Altogether, these data demonstrate that a small derepression of Ty1 transcription can result *per se* in a dramatic deregulation of Ty1 retrotransposition, highlighting the essential role of the Nup84 complex in keeping retrotransposons under tight transcriptional control.

## Discussion

Multiple systematic studies for regulators of the replication of LTR-retroelements in budding yeast had previously uncovered several NPC components [48–50,53], identifying in particular a post-transcriptional role for the nuclear basket [52]. In this report, we have focused on the contribution of a unique core sub-complex of the NPC, the Nup84 complex, to the expression and the retrotransposition of endogenous Ty elements. In the past, the expression of these repeated sequences might have been neglected in genome-wide analyses, mainly because reads that do not map to a unique locus are most often discarded. Here, our dedicated transcriptomic analysis pipeline has identified a shared function for most subunits of the Nup84 complex in the transcriptional repression of phylogenetically-distant Ty retroelements, including the most abundant and the most active Ty1 (Figs 1 and 2). When we further investigated the mechanism involved in this regulation, we highlighted that the Nup84 complex restricts Ty1 transcription through SUMOylation-dependent processes and not by modulating nuclear transport (Fig 3). We further scored an increase in Ty1 mobility in Nup84 complex mutants (Fig 4), in contrast with previous reports using alternative retrotransposition reporters [48–52] (S5 Fig). In particular, one of these earlier studies identified the *nup84Δ* mutant in a large-scale screen based on the observation of a decreased number of His^+^ papillae arising from retrotransposition events [49]. Yet, this phenotype was not validated in quantitative assays, a critical issue for such poorly-growing mutants [31,86]. Indeed, when we inactivated *NUP84* in the original strain transformed with an integrative plasmid carrying the Ty1-*his3AI* reporter [49,87], we quantified increased retrotransposition levels, in agreement with our data obtained with the chromosomal reporter (S5A and S5B Fig). However, our comparative assays with alternative reporters [48–52] generally indicated that the increased mobility scored in *nup* mutants (S5A–S5D Fig) was all the less pronounced that the reporter expression and transposition frequency were elevated in WT cells (S5E Fig). This is exemplified by Ty1 reporters under the control of the strong *GAL1* promoter [48,51], with which Nup84 complex mutants confirmedly displayed lower transposition frequencies (S5D Fig). This could similarly account for the unchanged Ty1 mobility previously scored in *nup* mutants using a centromeric plasmid-borne Ty1 reporter [52], which led to higher retrotransposition rates in WT cells as compared to our assay performed at 25°C (S5F Fig). In this view, the slight increase in retrotransposition that we scored in *nup* mutants carrying this reporter (S5C Fig) is not inconsistent with the previously described transposition frequencies [52]. Altogether, these reconciled data suggest that the Nup84 complex primarily represses Ty1 transcription, as revealed here with a Ty1-*his3AI* reporter naturally integrated in the genome. Yet, these nucleoporins become required for a post-transcriptional step of Ty1 replication cycle, when cells are artificially saturated with Ty1 transposons and/or when their transcriptional regulation is bypassed.

Although the rest of the transcriptome seems to be largely unaffected in Nup84 complex mutants, it is likely that other physiological or stress situations could lead to Nup84 complex-dependent changes in gene expression. Indeed, mechanism-wise, the common phenotypic signatures of Nup84 complex and *ulp1* mutants, as well as our epistasis analysis, support the view that the Nup84 complex represses Ty1 transcription by controlling the activity of the SUMO-deconjugating enzyme Ulp1 (Figs 3 and 4), which is itself sensitive to environmental conditions, alike Ty1 (*e*.*g*. chemical stresses, [88–90]). In this context, the regulation of Ty1 transcription through the Nup84 complex/Ulp1 axis echoes the previously reported impact of the nuclear basket in the repression of *GAL* genes [40]. For these loci, it was shown that the mislocalization of Ulp1 from the NPC resulted in down-regulation of the SUMOylation of the *GAL* promoter-bound transcriptional repressor Ssn6, triggering unscheduled gene derepression. With the large number of transcription factors known to regulate Ty1 promoters, many of them being SUMOylated (*e*.*g*. Gcr1, Tec1, Ste12, Gcn4, Rap1, Tye7; [91] and references therein), the Ty1 derepression scored in Nup84 complex or *ulp1* mutants probably originates from a combination of SUMO-dependent regulations for multiple targets in the transcriptional machinery, their expression levels being unaffected in *nup* mutants (S1 Table). Since exposure of yeast cells to DNA-damaging agents also activates the transcription of Ty1 [92–95], changes in the SUMOylation and the activity of DNA damage response factors, as reported in Nup84 complex and *ulp1* mutants [31], could similarly stem for Ty1 derepression. Still, the phenotypes we observed with other mutants involved in SUMO conjugation suggest that it is the decreased SUMOylation of such yet-to-be-identified factors that ultimately leads to the activation of Ty1.

While Ty1 derepression could be triggered by a nucleoplasmic fraction of Ulp1, the proximity of Ty1 sequences with Ulp1 SUMO-deconjugating activity bound to the NPC could activate Ty1 transcription in a spatially-regulated manner, as proposed for *GAL* genes. Furthermore, beside their impact on the activity of transcription factors, SUMOylation events could also trigger the recruitment of Ty1 elements to the NPC, as established for the inducible *GAL1-10* and *INO1* loci [40,69]. In this respect, it is striking that *tDNA* loci, which are adjacent to most Ty1 sequences in the genome due to Ty1 preferential integration close to Pol III-transcribed genes (reviewed in [96]), undergo cell-cycle dependent relocalization to the NPCs [42], and that a number of transcription factors, including the *bona fide* Ty1 activators Gcn4 and Ste12, are sufficient to promote the NPC association of their target sequences [97]. At this position, Ty1 elements may integrate the signals from various NPC-associated factors, including the Mediator co-activator complex [14], which also acts at Ty1 promoters [98], or specific partners of the Nup84 complex, as suggested by the co-evolution scored between Ty1 elements and *NUP84* sequences in *Saccharomyces* yeasts [51]. In the future, genome-wide and imaging analysis investigating the position of Ty elements in the nucleus will be instrumental to decipher the relationships between the expression of transposable elements and the dynamics of their localization with respect to NPCs during the course of the cell cycle. These additional studies should also point whether distinct mechanisms are at play for *Copia* (Ty1, Ty2) and *Gypsy* (Ty3) retroelements that are similarly derepressed in *nup* mutants.

With their specific effect on Ty1 transcription, Nup84 complex mutants provide a unique tool to decipher the relationships between Ty1 RNA production and retrotransposition (Fig 4). While Ty1 RNAs are confirmed to be very abundant RNAP II transcripts in our transcriptomic data (Fig 1), in agreement with earlier estimates [45,46], they appear to be limiting for reverse transcription, as revealed by the exponential increase in cDNA amounts driven by RNA accumulation (Fig 4F). Solving this apparent paradox will definitely require to identify which host-specific factors limit cDNA levels in cells, from their synthesis in the cytoplasmic virus-like particles, whose accumulation could favor reverse transcription, to their integration into the nuclear genome. Similarly, these changes in cDNA amounts have even more pronounced consequences on Ty1 mobility (Fig 4H), suggesting that cDNA molecules are also limiting for retrotransposition. This observation implies that while many controls are effective in limiting the retrotransposition of Ty1 under normal growth conditions, they can be easily overridden by activating Ty transcription, especially under adverse conditions, when retrotransposition activity contributes to the response to stress. Altogether, our results thus highlight the importance of keeping the transcription of Ty elements at a level that would prevent their excessive propagation in the genome. The pivotal role of the Nup84 complex in this process thereby adds up to the ever-growing reported mechanisms that silence TE transcription in eukaryotes, including DNA methylation, chromatin repressive marks and RNA interference [99].

## Material & methods

### Plasmids, yeast strains and growth

All the strains used in this study are haploid and isogenic to BY4741/2 and are listed in S2 Table. Yeast cells were grown in standard yeast extract peptone dextrose (YPD) or synthetic complete (SC) media lacking appropriate amino acids. Unless indicated, yeast cells were grown at 25°C. Growth assays were performed by spotting serial dilutions of exponentially-growing cells on solid YPD medium and incubating the plates at the indicated temperatures. All plasmids and primers used in this study are reported in S3 and S4 Tables. Independent sets of *nup* mutant strains, where all non-essential subunits of the Nup84 complex were individually inactivated in two distinct backgrounds, were assessed.

### Gene expression analyses

RNA quantification and RNA polymerase II chromatin immunoprecipitation were performed as described in [89], except that cells were systematically grown at 25°C. Briefly, total RNAs were extracted from yeast cells disrupted by bead beating and purified using the Nucleospin RNA II kit (Macherey-Nagel). For RNA-seq analysis, stranded libraries were prepared after ribosomal RNA removal and further sequenced on an Illumina NovaSeq paired-end sequencing platform with 150 bp read length by Novogene (UK) Company Limited. For RT-qPCR analysis, total RNAs were reverse transcribed with Superscript-II reverse transcriptase (Thermo Fisher Scientific) and cDNAs were quantified by real-time PCR with a QuantStudio 5 system using the Power Track SYBR Green Master mix (Thermo Fisher Scientific) and the primers listed in S4 Table. The amounts of the RNAs of interest were normalized relative to *ACT1* mRNA values (invariant in our transcriptomic analyses), and further set to 1 for WT cells. Unless indicated, values obtained for the Ty1mid amplicon (Fig 2B) were displayed. RNA polymerase II distribution along the genes of interest was determined by ChIP as previously reported [89]. Input and immunoprecipitated DNA amounts were quantified by real-time PCR as above.

### Western blot analysis

Total protein extraction from yeast cells was performed by the NaOH–TCA lysis method [100]. Samples were separated on 4–12% SDS-PAGE gels and transferred to nitrocellulose membranes, or PVDF for SUMO detection. Western blot analysis was performed using the following antibodies: polyclonal anti-VLP (to detect p49/p45-Gag polypeptides [93]; 1:10,000), polyclonal anti-Integrase (8B11, a gift from J. Boeke; 1:100), polyclonal anti-p18/p22 ([61]; 1:5,000, a gift from D. Garfinkel), polyclonal anti-SUMO ([36]; 1:2,000) and monoclonal anti-Pab1 (Abcam; 1:1,000). Total proteins were detected upon coupling with stain-free fluorescent reagents in TGX precast gels (Mini-PROTEAN, Bio-Rad), using a ChemiDOC imager (Bio-Rad) according to the manufacturer’s instructions. Quantification of signals was performed using the ImageJ software.

### cDNA quantification

Ty1 cDNA quantification assay was adapted from [73–75]. Briefly, genomic DNA (gDNA) was phenol-extracted from exponentially-growing yeast cells after cell wall disruption by digestion with zymolyase (100T; Fisher Scientific), and further isolated by ethanol precipitation. After quantification with Qubit dsDNA BR assay kit (Thermo Fisher Scientific), 1μg of gDNA was digested by the MssI restriction enzyme to generate blunt-ended fragments, similar to Ty1 cDNA ends (FastDigest enzyme; Thermo Fisher Scientific) for 2h at 37°C in a total volume of 20μL, conditions in which a complete digestion was observed. 100ng of digested gDNA were then ligated for 1 hour at 22°C with 600ng adapters (generated by annealing oligos O-AMA158 and O-AMA159; S4 Table), in the presence of 2.5 units of T4 DNA ligase (Thermo Fisher Scientific). The reaction was then stopped by incubation at 65°C for 10min. cDNA quantification by real-time PCR was performed on a 20-fold dilution of the ligation mixture with a QuantStudio 5 system using the LC480 SYBR Green Master mix (Roche) and primers hybridizing in the adapter and Ty1 LTR (S4 Table). The cDNA amounts were normalized relative to the number of genomic copies, which were evaluated by quantifying the amount of a specific MssI-digested locus on chromosome I, and further set to 1 for WT cells.

### Retrotransposition assay

Ty1 mobility was measured as previously described in [101]. Briefly, four independent clones of strains harboring a Ty1-*his3AI* chromosomal reporter were grown to saturation for 24h at 30°C in liquid YPD. Each culture was diluted thousand-fold in 2x 1mL of liquid YPD and grown for 3 days to saturation at 25°C. Aliquots of cultures were plated on YPD (2x 100μL of a 1:20,000 dilution) and SC-HIS (2x 1ml). Plates were incubated for 4 days at 30°C and colonies were counted to determine the fraction of [HIS+] prototrophs. A retrotransposition frequency was thus calculated as the median of the ratios of number of [HIS+] cells to viable cells for each of the four independent clones. Retrotransposition frequencies were then defined as the mean of at least three medians. Similar experiments were performed from four independent clones transformed with pCenTy or pGal-Ty1 plasmids, except that clones were cultured for 24h at 30°C in SC-URA or GGL-URA (GGL: 0.17% YNB, 0.5% ammonium sulfate, 0.05% glucose, 2% lactate and 2% glycerol) liquid media, respectively, and then diluted for 3 days at 25°C in SC-URA or GGL-URA + 2% Galactose, respectively.

### Bioinformatic analyses of RNA-seq data

Analyses were performed using Galaxy (https://usegalaxy.org/). Paired-end raw reads were trimmed by the Trimmomatic tool (version 0.38.0) [102] and mapped to SacCer3 genome using HISAT2 (version 2.1.0) [103], yielding 96–98% mapped reads. Only reads properly paired were kept for further analysis (SAMtools version1.9 [104]). Then, read counts for each genomic feature was calculated by featureCounts by assigning fractions to multi-mapping reads (version 1.6.4) [105] and differential expression analysis was performed using DESeq2 (version 2.11.40.6) [106]. To restrict the analysis to single-mapping reads, the featureCounts tool was used upon filtering out reads with MAPQ score below 5. This filter removed all the reads multi-mapping to distinct Ty loci (80%, 80% and 30% of total Ty1, Ty2 and Ty3 mappers, respectively). Gene Ontology analysis was performed on differently-expressed genes (FC>2) using the GO Slim Mapper tool available in Saccharomyces Genome Database (https://www.yeastgenome.org/goSlimMapper).

For metagene analysis, the aligned reads files (BAM) were first converted to normalized (RPKM) stranded coverage files (bigWig) with 1 bp bins using bamCoverage (deepTools version 3.3.2) [107]. The mean coverage was then obtained from the three replicates of each strain using bigwigCompare (deepTools). Metaplots were finally generated with computeMatrix and plotProfile (deepTools), representing the mean and standard error of read coverage over the 32 Ty1 genomic copies for each strain.

Distance to the closest *tDNA* was determined for each Ty1 copy using ClosestBed from bedtools (version 2.29.0) [108]. The chromosomal position of each Ty1 copy was determined relative to the centromere (set to 0) and the telomere (set to 1) using the calculation [distance between 5’ LTR and centromere/length of chromosome arm].

### Statistical analyses

The following statistical tests were used: a two-sided Wilcoxon rank-sum test was used to compare the median log_2_(FC) of a dataset with a hypothetical median of 0 (Figs 1C and 3G) and a Mann-Whitney test was used in S1F Fig. Alternatively, the two-sided Welch’s t test was used allowing unequal variance (other figures). * p <0.05; ** p <0.01; *** p <0.001; **** p <0.001; ns, not significant. Statistical tests were performed using GraphPad Prism version 9.0.0.

## Supporting information

S1 Fig Control experiments, related to transcriptome and reporter analyses. **(A)** Characterization of the non-essential deletion mutants of the Nup84 complex. Serial 5-fold dilutions of WT and mutant cells were grown at the indicated temperatures on YPD solid medium. **(B)** Reproducibility of RNA levels measurements in RNA-sequencing data across biological replicates. RNA levels are represented as log_2_ (FPKM). The Spearman’s correlation coefficient is indicated for each pair of replicates. **(C)** Ty1, Ty2 and Ty3 RNA levels in WT and *spt3Δ* cells, as measured by RT-qPCR (mean±SD, n = 3, relative to WT and normalized to *ACT1* mRNA values). ns, not significant; * p<0.05; ** p<0.01, Welch’s t test with comparison to the WT strain. **(D)** Total Ty1 RNA levels in the different Ty1-*lacZ* fusion strains used in Fig 1J, as measured by RT-qPCR (mean±SD, n = 3, relative to WT BL-*lacZ* and normalized to *ACT1* mRNAs). **(E)** Location of the Ty1-*lacZ* fusions on chromosomes (represented to scale). Ty1 are represented by a full or hatched grey line, depending on their location on the Watson or Crick DNA strands, respectively. The presence of *tDNA*s in the close proximity of the Ty1*-lacZ* fusions is indicated. **(F)** The 32 Ty1 genomic copies were ranked in four distinct quartiles (Q1 to Q4) according to their log_2_ Fold change in *nup133Δ* or *nup84Δ* mutants relative to WT cells. From top to bottom, log_2_ Fold changes (relative to WT), basal Ty1 RNA levels (fragments per kb per million reads mapped [FPKM] in WT RNA-seq data), distances from the closest *tDNA* gene (bp) and chromosomal positions (with respect to centromeres, set to 0, and telomeres, set to 1) were further represented as box-plots for each quartile. ns, not significant; * p<0.05; *** p<0.001, Mann-Whitney test.
(TIF)

S2 Fig Control experiments, related to RNAP II ChIP and Ty1 promoter analyses. **(A)** RNAP II occupancy on all Ty1 genomic copies as determined by ChIP in WT, *nup133Δ* and *nup84Δ* cells. Values (mean±SD, n≥3) are expressed as a percentage of IP and normalized to a distinct intergenic region from Fig 2A. **(B-C)** RNAP II occupancy on the intergenic region #1 [111] **(B)** and intergenic region #2 [112] **(C)** as determined by ChIP in WT, *nup133Δ* and *nup84Δ* cells. Values (mean±SD, n≥3) are expressed as a percentage of IP. **(D)** Whole cell extracts of the indicated strains analyzed by western blotting using anti-VLP antibodies, revealing p49/p45-Gag proteins, and anti-p18 antibodies, revealing p18- and p22-Gag species. p18 likely arises from p22 processing as previously described [61]. Molecular weights are indicated (kDa). **(E)** Quantification of p49/p45-Gag and p18/p22-Gag levels from (D) represented as the ratio of p18/p22 over p49/p45-Gag protein levels (mean±SD, n = 3, relative to WT). ns, not significant; * p<0.05; ** p<0.01, Welch’s t test with comparison to the WT strain.
(TIF)

S3 Fig The Nup84 complex and Ulp1 do not interfere with the expression of Ty regulators but rather impact SUMOylation processes. **(A)** Venn diagrams indicating the overlap between genes commonly up-regulated (*upper panel*) or down-regulated (*lower panel*) in *nup133Δ*, *nup84Δ* and *ulp1* mutants and Ty1 Retromobility Host Factor (RHF) or Restrictor of Ty Transposition (RTT) genes listed in [47], respectively. Among the deregulated genes in *nup133Δ*, *nup84Δ* and *ulp1* mutants, all genes corresponding to the indicated GO Slim terms are listed. **(B)** Ty1 RNA levels in WT cells and deletants of the genes listed in (A), as measured by RT-qPCR (mean±SD, n = 3, relative to WT and normalized to *ACT1* mRNAs). Ty1 RNA levels in the *nup84Δ* mutant are shown as a control of Ty1 induction. ns, not significant; * p<0.05, Welch’s t test with comparison to the WT strain. **(C)** Whole cell extracts of the indicated strains analyzed by western blotting using anti-SUMO antibodies, revealing the pattern of SUMO-conjugates (*top panel*) and free, non-conjugated SUMO (*mid panel*). Total proteins were detected with the stain-free methodology (*bottom panel*) and molecular weights are indicated (kDa). Note the impaired SUMO processing in the *ulp1* mutant, as revealed by the presence of a slower migrating form of free SUMO, and the strong overall decrease in SUMO-conjugation in *ubc9-1* cells. The total protein image in the right panel is the same as in Fig 4B since the same samples were used for both figures.
(TIF)

S4 Fig Analysis of the retrotransposition cycle in *nup* and *ulp1* mutants. **(A)** *Left panel*: Whole cell extracts of the indicated strains analyzed by western blotting using anti-VLP antibodies, revealing Gag proteins. Pab1 is used as a loading control. Molecular weights are indicated (kDa). *Mid panel*: Quantification of Gag levels from western blot analyses (mean±SD, n = 2, relative to WT and normalized to Pab1 protein levels). *Right panel*: Whole cell extracts of the indicated strains analyzed by western blotting using anti-integrase antibodies, revealing all Pol maturation intermediates leading to integrase (p71-IN). Pab1 is used as a loading control. Molecular weights are indicated (kDa). Intervening lanes that were spliced out are indicated by dotted lines. **(B)** Total Ty1 RNA levels in WT cells and non-essential mutants of the Nup84 complex (*left panel*) or *ΔN-ulp1* mutant (*right panel*), as measured by RT-qPCR (mean±SD, n≥3, relative to WT and normalized to *ACT1* mRNAs). The *spt3Δ* mutant is used as a control for qPCR specificity because Ty1 expression is strongly decreased in this mutant. **(C)** Principle of Ty1 cDNA quantification. Adapters (in red) are ligated to both ends of non-integrated Ty1 cDNA molecules and to every genomic locus arising from MssI blunt-end digestion. qPCR amplicons used for cDNA detection and for normalization to a MssI restriction fragment from Chromosome I are indicated in black. One primer hybridizes in the adapter sequence while the other primer is specific of either Ty1 LTR or the genomic locus. **(D)** Total Ty1 cDNA levels in WT cells and non-essential mutants of the Nup84 complex, as measured by qPCR (mean±SD, n = 4, relative to WT and normalized to the values of a non-digested genomic locus, *ACT1*). The *spt3Δ* mutant and a ligation reaction performed without adapters are used as a control for qPCR specificity to demonstrate that Ty1 genomic copies were not detected by this assay. **(E)** Comparison of Ct values obtained from qPCR amplicons detecting Ty1 cDNA molecules and a genomic locus of the chromosome I or the chromosome XIV previously digested by MssI. Values and medians of 7 different experiments are plotted. **(F)** Total Ty1 cDNA levels in WT cells and *fus3Δ* or *rrm3Δ* mutant, as measured by qPCR (mean±SD, n = 3, relative to WT and normalized to the values of a chromosome I genomic locus digested by MssI). The *spt3Δ* mutant is used as a control for qPCR specificity because Ty1 cDNAs are strongly decreased in this mutant (same values as Fig 4E). Previous Ty1 cDNA levels measurements obtained by Southern blot are indicated in brackets (a, from [81]; b, from [78]; c, from [80]) **(G)** Ty1-*his3AI* RNA levels in WT cells and non-essential mutants of the Nup84 complex (*left panel*) or *ΔN-ulp1* mutant (*mid panel*) or *mlp1Δ mlp2Δ* mutant (*right panel*), as measured by RT-qPCR (mean±SD, n≥3, relative to WT and normalized to *ACT1* mRNAs). The *spt3Δ* mutant is used as a control for qPCR specificity, as above. ns, not significant; * p<0.05; ** p<0.01; *** p<0.001, Welch’s t test with comparison to the WT strain.
(TIF)

S5 Fig Comparison of retrotransposition frequencies using distinct Ty1 reporters in *nup* mutants. **(A-D)** Retrotransposition frequencies (log scale, mean±SD, n≥3) of Ty1-*his3AI* reporters in WT, *nup133Δ* and *nup84Δ* cells. **(A)** Values obtained with the Ty1-*his3AI* chromosomal reporter (also featured in Fig 4G). **(B)** Values obtained with the Ty1-*his3AI* reporter carried on the pBJC573 integrative plasmid and under the control of its own promoter (DG2122 derived strains, as previously used in [49,87]). **(C)** Values obtained with the Ty1-*his3AI* reporter carried on a centromeric plasmid and under the control of its own promoter (pCenTy, as previously used in [52]). **(D)** Values obtained with the Ty1-*his3AI* reporter carried on a multicopy plasmid and under the control of the inducible *GAL1* promoter (pGal-Ty1-*his3AI*). Note that *GAL1* promoter-driven reporters have been previously used in [48,51]. **(E)** Comparison of Ty1-*his3AI* RNA levels (log scale, relative to *ACT1* mRNAs, mean±SD, n≥4) and retrotransposition frequencies (log scale, mean±SD, n≥4, same values as panels A-D) in WT cells for the aforementioned reporters. The dashed line indicates the RNA levels of the highly-expressed endogenous Ty1(LR4) element (quantified in the LR4-*lacZ* strain). Note that in view of its expression levels, the chromosomal Ty1-*his3AI* reporter appears to be the best proxy for endogenous Ty1 activity. **(F)** Comparison between retrotransposition frequencies (log scale, mean±SD) obtained in WT, *nup133Δ* and *nup84Δ* cells carrying the centromeric Ty1-*his3AI* reporter in this study (same values as in S5C) and in a previous report [52]. ns, not significant; * p<0.05; ** p<0.01; *** p<0.001, Welch’s t test.
(TIF)

S1 Table Genes commonly affected in Nup84 complex / *ulp1* mutants. The genes commonly up-regulated (log_2_(FC)>1) or down-regulated (log_2_(FC)<-1) in *nup133Δ*, *nup84Δ* and *ulp1* mutants are listed. Log_2_(FC) and adjusted p-values obtained following the DEseq analysis are indicated for each mutant. Candidate genes involved in gene expression largo sensu are highlighted in yellow together with the associated GO Slim categories.
(XLSX)

S2 Table Yeast strains used in this study. (DOCX)

S3 Table Plasmids used in this study. (DOCX)

S4 Table Primers used in this study. (DOCX)

S1 Dataset Numerical data and summary statistics of graphs. (XLSX)
