# Supplementary figures and images for: A nuclear pore sub-complex restricts the propagation of Ty retrotransposons by limiting their transcription
Source: PLoS Genet. 2021 Nov 1;17(11):e1009889. doi: 10.1371/journal.pgen.1009889 (PMC8585004; doi:10.1371/journal.pgen.1009889)

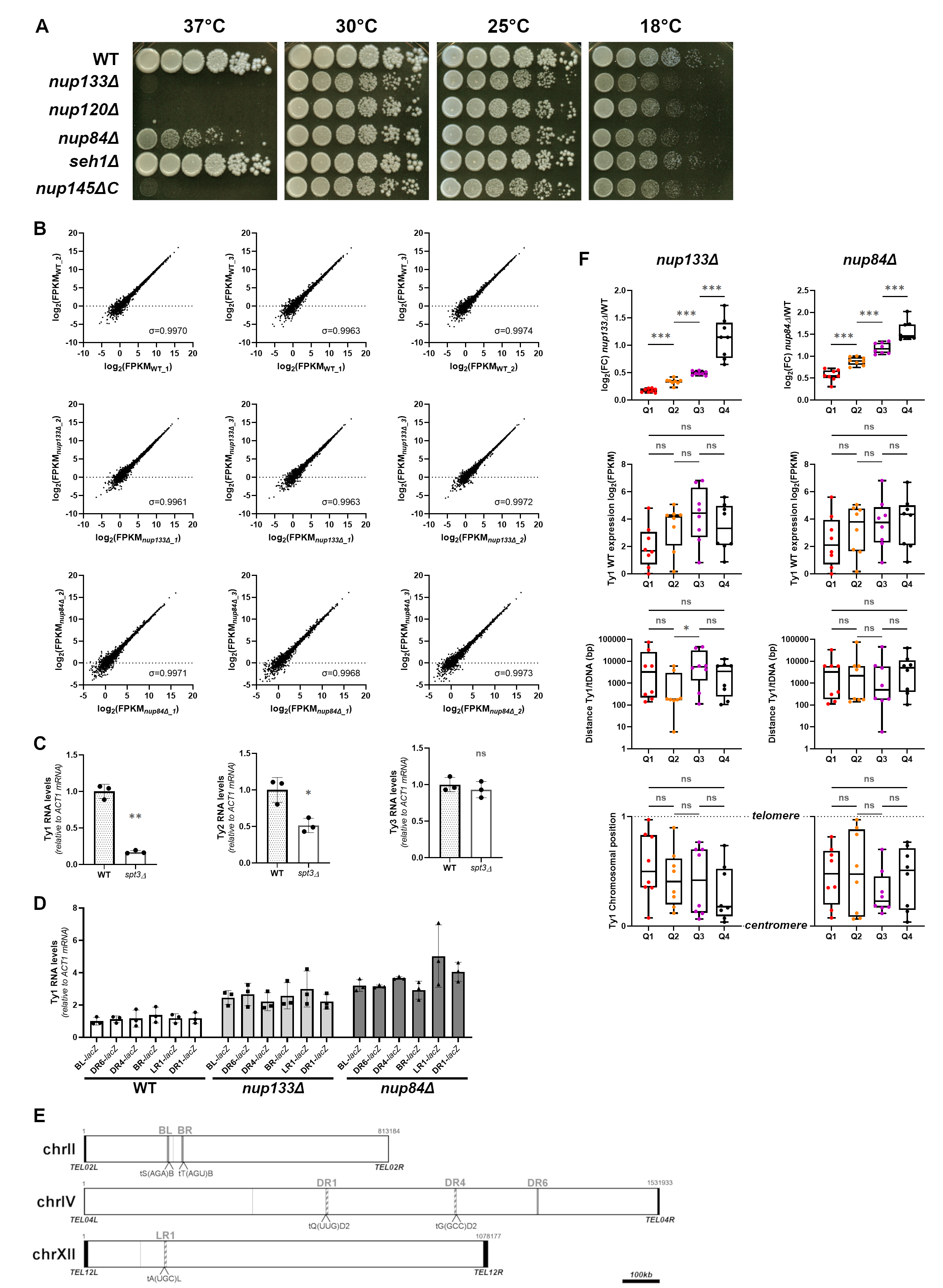

Supplement: S1 Fig — (A) Characterization of the non-essential deletion mutants of the Nup84 complex. Serial 5-fold dilutions of WT and mutant cells were grown at the indicated temperatures on YPD solid medium. (B) Reproducibility of RNA levels measurements in RNA-sequencing data across biological replicates. RNA levels are represented as log2 (FPKM). The Spearman’s correlation coefficient is indicated for each pair of replicates. (C) Ty1, Ty2 and Ty3 RNA levels in WT and spt3Δ cells, as measured by RT-qPCR (mean±SD, n = 3, relative to WT and normalized to ACT1 mRNA values). ns, not significant; * p<0.05; ** p<0.01, Welch’s t test with comparison to the WT strain. (D) Total Ty1 RNA levels in the different Ty1-lacZ fusion strains used in Fig 1J, as measured by RT-qPCR (mean±SD, n = 3, relative to WT BL-lacZ and normalized to ACT1 mRNAs). (E) Location of the Ty1-lacZ fusions on chromosomes (represented to scale). Ty1 are represented by a full or hatched grey line, depending on their location on the Watson or Crick DNA strands, respectively. The presence of tDNAs in the close proximity of the Ty1-lacZ fusions is indicated. (F) The 32 Ty1 genomic copies were ranked in four distinct quartiles (Q1 to Q4) according to their log2 Fold change in nup133Δ or nup84Δ mutants relative to WT cells. From top to bottom, log2 Fold changes (relative to WT), basal Ty1 RNA levels (fragments per kb per million reads mapped [FPKM] in WT RNA-seq data), distances from the closest tDNA gene (bp) and chromosomal positions (with respect to centromeres, set to 0, and telomeres, set to 1) were further represented as box-plots for each quartile. ns, not significant; * p<0.05; *** p<0.001, Mann-Whitney test. (TIF) [file pgen.1009889.s001.tif]

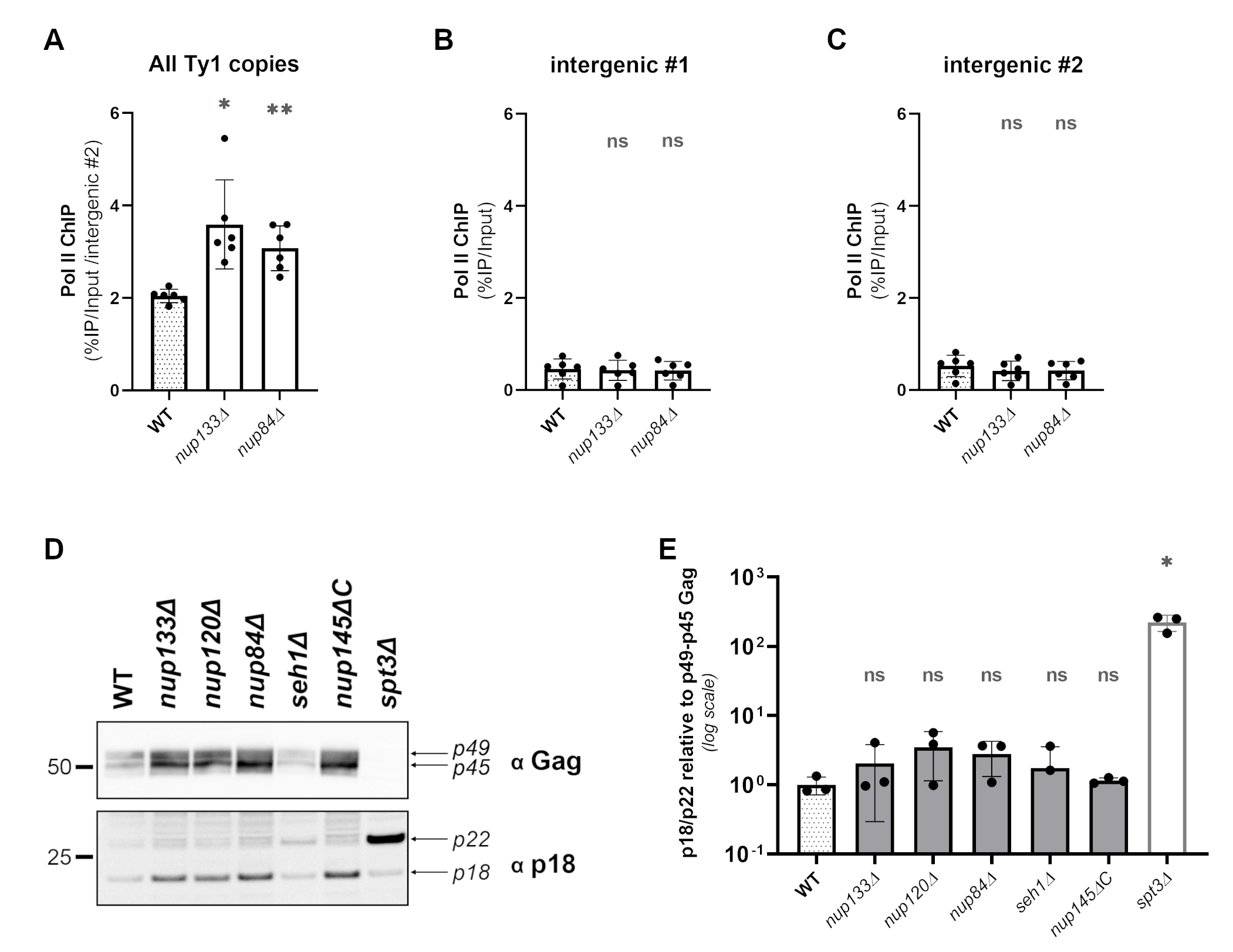

Supplement: S2 Fig — (A) RNAP II occupancy on all Ty1 genomic copies as determined by ChIP in WT, nup133Δ and nup84Δ cells. Values (mean±SD, n≥3) are expressed as a percentage of IP and normalized to a distinct intergenic region from Fig 2A. (B-C) RNAP II occupancy on the intergenic region #1 [111] (B) and intergenic region #2 [112] (C) as determined by ChIP in WT, nup133Δ and nup84Δ cells. Values (mean±SD, n≥3) are expressed as a percentage of IP. (D) Whole cell extracts of the indicated strains analyzed by western blotting using anti-VLP antibodies, revealing p49/p45-Gag proteins, and anti-p18 antibodies, revealing p18- and p22-Gag species. p18 likely arises from p22 processing as previously described [61]. Molecular weights are indicated (kDa). (E) Quantification of p49/p45-Gag and p18/p22-Gag levels from (D) represented as the ratio of p18/p22 over p49/p45-Gag protein levels (mean±SD, n = 3, relative to WT). ns, not significant; * p<0.05; ** p<0.01, Welch’s t test with comparison to the WT strain. (TIF) [file pgen.1009889.s002.tif]

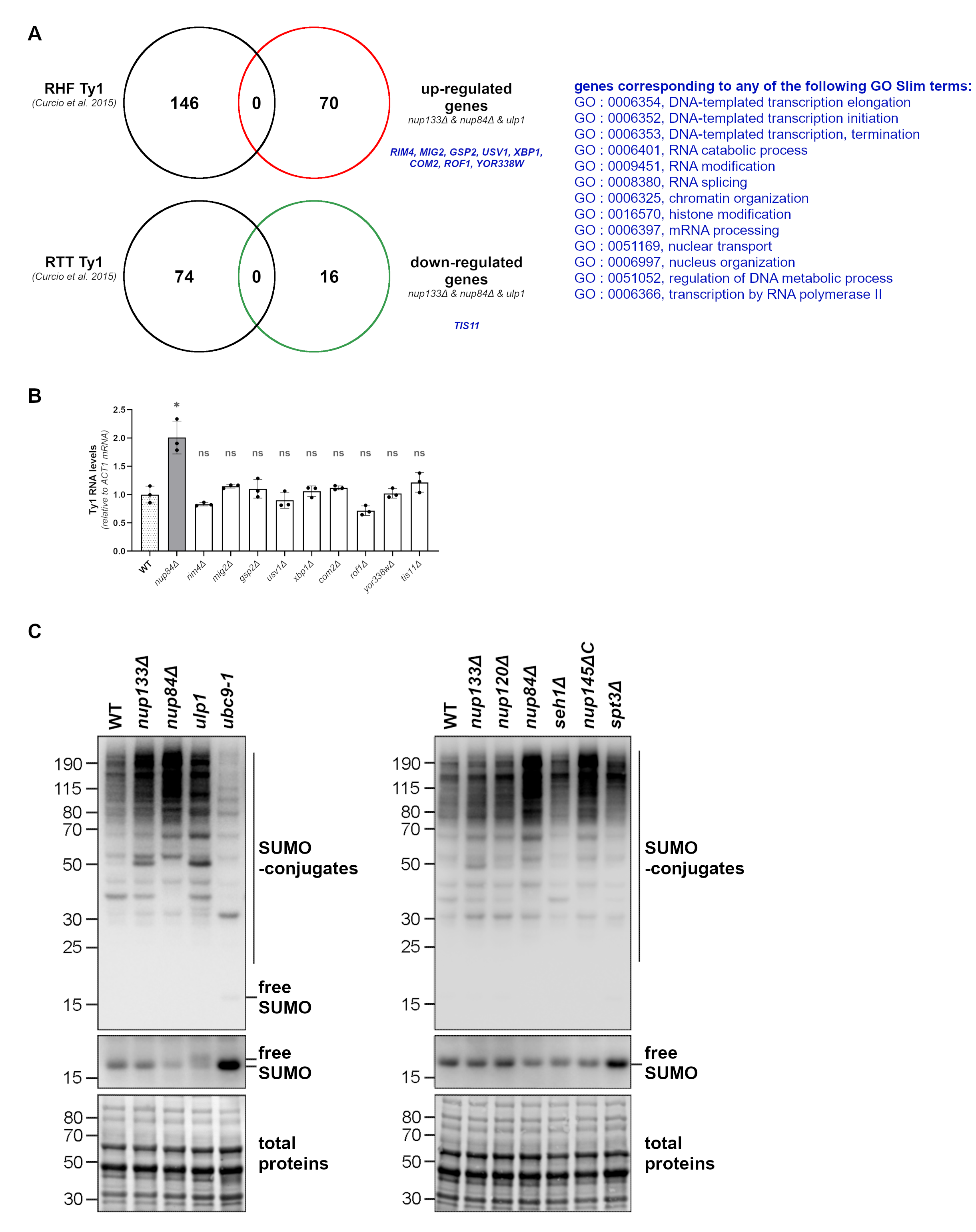

Supplement: S3 Fig — (A) Venn diagrams indicating the overlap between genes commonly up-regulated (upper panel) or down-regulated (lower panel) in nup133Δ, nup84Δ and ulp1 mutants and Ty1 Retromobility Host Factor (RHF) or Restrictor of Ty Transposition (RTT) genes listed in [47], respectively. Among the deregulated genes in nup133Δ, nup84Δ and ulp1 mutants, all genes corresponding to the indicated GO Slim terms are listed. (B) Ty1 RNA levels in WT cells and deletants of the genes listed in (A), as measured by RT-qPCR (mean±SD, n = 3, relative to WT and normalized to ACT1 mRNAs). Ty1 RNA levels in the nup84Δ mutant are shown as a control of Ty1 induction. ns, not significant; * p<0.05, Welch’s t test with comparison to the WT strain. (C) Whole cell extracts of the indicated strains analyzed by western blotting using anti-SUMO antibodies, revealing the pattern of SUMO-conjugates (top panel) and free, non-conjugated SUMO (mid panel). Total proteins were detected with the stain-free methodology (bottom panel) and molecular weights are indicated (kDa). Note the impaired SUMO processing in the ulp1 mutant, as revealed by the presence of a slower migrating form of free SUMO, and the strong overall decrease in SUMO-conjugation in ubc9-1 cells. The total protein image in the right panel is the same as in Fig 4B since the same samples were used for both figures. (TIF) [file pgen.1009889.s003.tif]

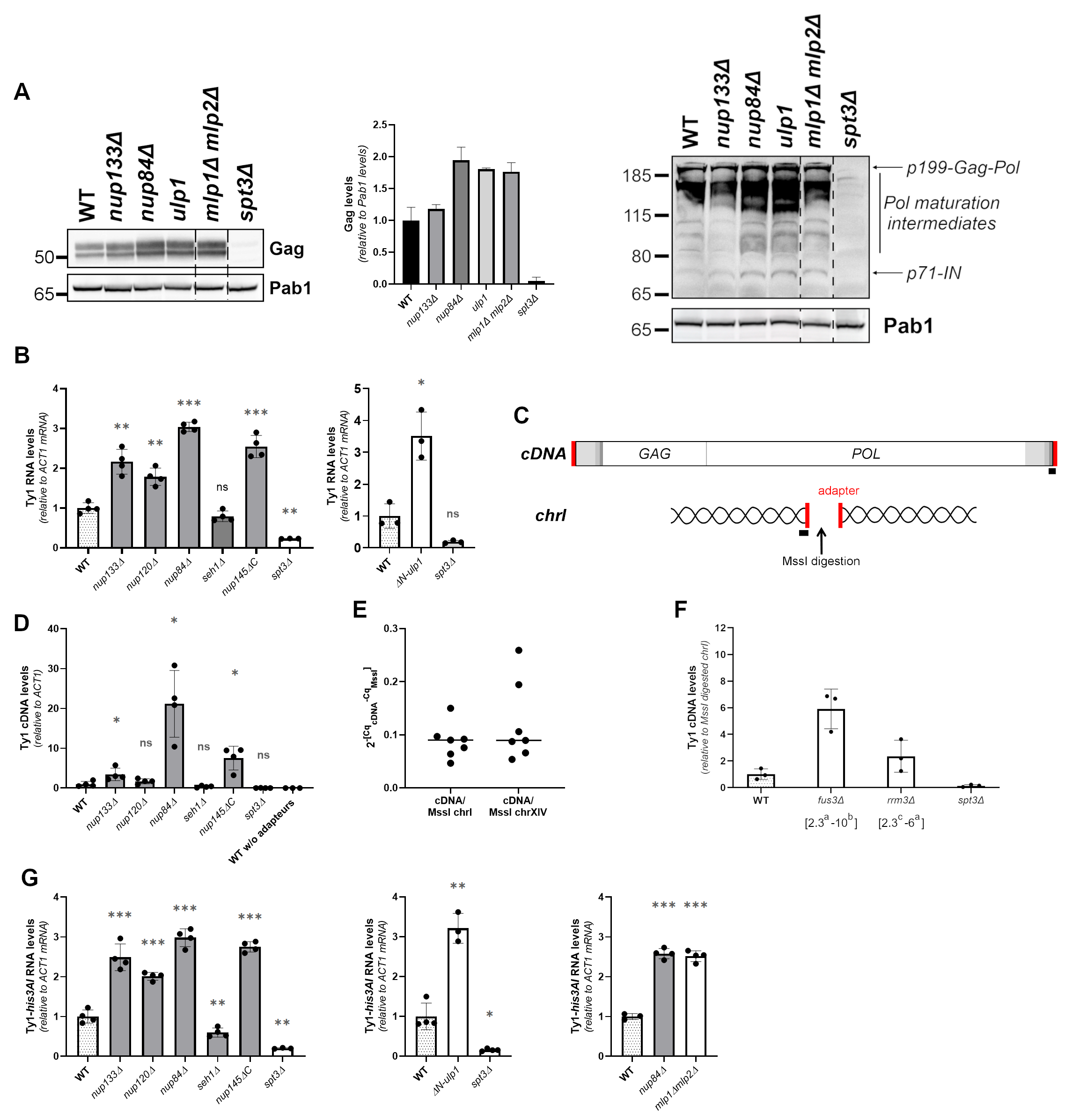

Supplement: S4 Fig — (A) Left panel: Whole cell extracts of the indicated strains analyzed by western blotting using anti-VLP antibodies, revealing Gag proteins. Pab1 is used as a loading control. Molecular weights are indicated (kDa). Mid panel: Quantification of Gag levels from western blot analyses (mean±SD, n = 2, relative to WT and normalized to Pab1 protein levels). Right panel: Whole cell extracts of the indicated strains analyzed by western blotting using anti-integrase antibodies, revealing all Pol maturation intermediates leading to integrase (p71-IN). Pab1 is used as a loading control. Molecular weights are indicated (kDa). Intervening lanes that were spliced out are indicated by dotted lines. (B) Total Ty1 RNA levels in WT cells and non-essential mutants of the Nup84 complex (left panel) or ΔN-ulp1 mutant (right panel), as measured by RT-qPCR (mean±SD, n≥3, relative to WT and normalized to ACT1 mRNAs). The spt3Δ mutant is used as a control for qPCR specificity because Ty1 expression is strongly decreased in this mutant. (C) Principle of Ty1 cDNA quantification. Adapters (in red) are ligated to both ends of non-integrated Ty1 cDNA molecules and to every genomic locus arising from MssI blunt-end digestion. qPCR amplicons used for cDNA detection and for normalization to a MssI restriction fragment from Chromosome I are indicated in black. One primer hybridizes in the adapter sequence while the other primer is specific of either Ty1 LTR or the genomic locus. (D) Total Ty1 cDNA levels in WT cells and non-essential mutants of the Nup84 complex, as measured by qPCR (mean±SD, n = 4, relative to WT and normalized to the values of a non-digested genomic locus, ACT1). The spt3Δ mutant and a ligation reaction performed without adapters are used as a control for qPCR specificity to demonstrate that Ty1 genomic copies were not detected by this assay. (E) Comparison of Ct values obtained from qPCR amplicons detecting Ty1 cDNA molecules and a genomic locus of the chromosome I or the chromos [file pgen.1009889.s004.tif]

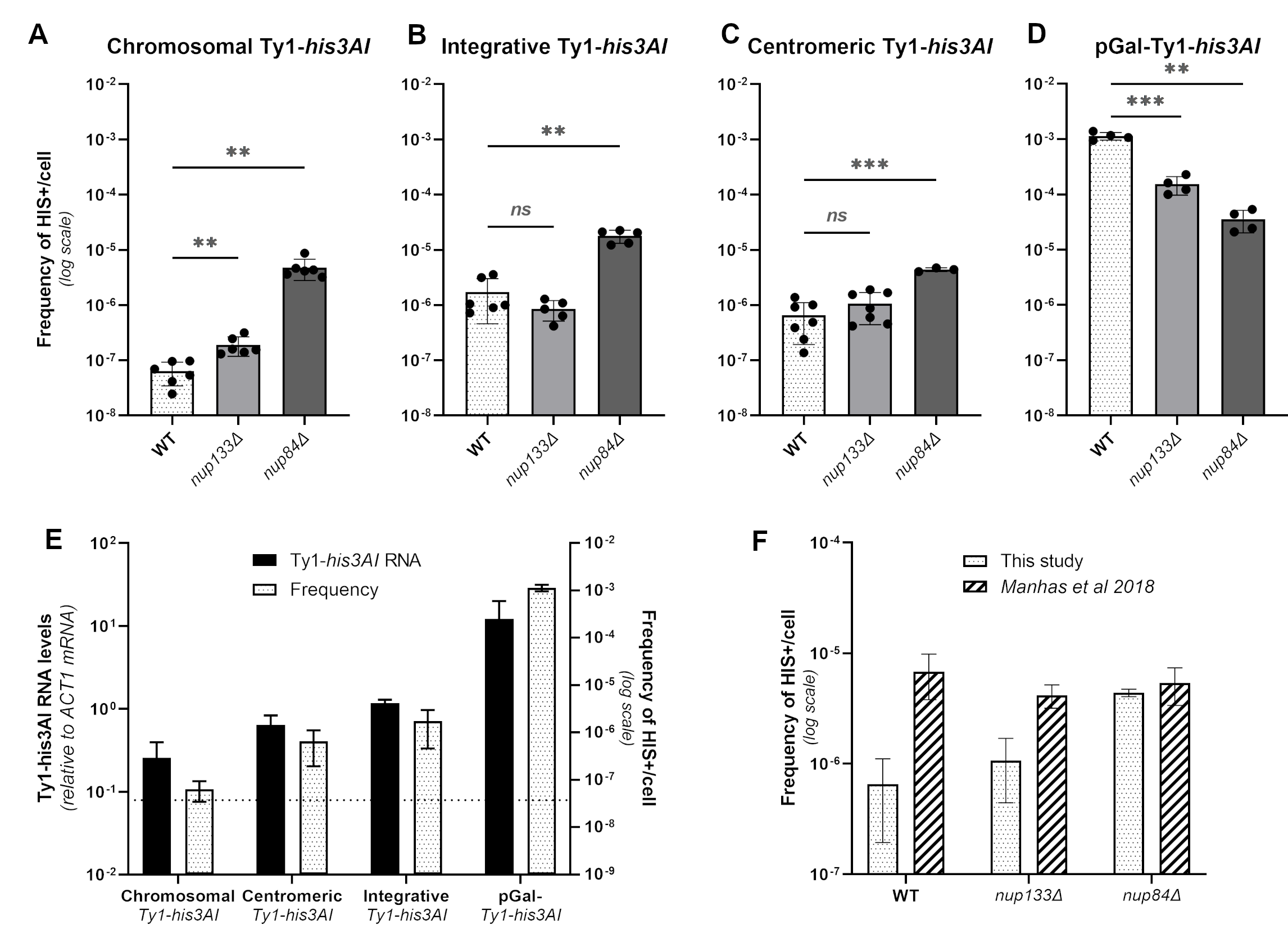

Supplement: S5 Fig — (A-D) Retrotransposition frequencies (log scale, mean±SD, n≥3) of Ty1-his3AI reporters in WT, nup133Δ and nup84Δ cells. (A) Values obtained with the Ty1-his3AI chromosomal reporter (also featured in Fig 4G). (B) Values obtained with the Ty1-his3AI reporter carried on the pBJC573 integrative plasmid and under the control of its own promoter (DG2122 derived strains, as previously used in [49,87]). (C) Values obtained with the Ty1-his3AI reporter carried on a centromeric plasmid and under the control of its own promoter (pCenTy, as previously used in [52]). (D) Values obtained with the Ty1-his3AI reporter carried on a multicopy plasmid and under the control of the inducible GAL1 promoter (pGal-Ty1-his3AI). Note that GAL1 promoter-driven reporters have been previously used in [48,51]. (E) Comparison of Ty1-his3AI RNA levels (log scale, relative to ACT1 mRNAs, mean±SD, n≥4) and retrotransposition frequencies (log scale, mean±SD, n≥4, same values as panels A-D) in WT cells for the aforementioned reporters. The dashed line indicates the RNA levels of the highly-expressed endogenous Ty1(LR4) element (quantified in the LR4-lacZ strain). Note that in view of its expression levels, the chromosomal Ty1-his3AI reporter appears to be the best proxy for endogenous Ty1 activity. (F) Comparison between retrotransposition frequencies (log scale, mean±SD) obtained in WT, nup133Δ and nup84Δ cells carrying the centromeric Ty1-his3AI reporter in this study (same values as in S5C) and in a previous report [52]. ns, not significant; * p<0.05; ** p<0.01; *** p<0.001, Welch’s t test. (TIF) [file pgen.1009889.s005.tif]
