# Supplementary material for: A nuclear pore sub-complex restricts the propagation of Ty retrotransposons by limiting their transcription
Source: PLoS Genet. 2021 Nov 1;17(11):e1009889. doi: 10.1371/journal.pgen.1009889 (PMC8585004; doi:10.1371/journal.pgen.1009889)
Supplement: S2 Table — (DOCX) [file pgen.1009889.s007.docx]

**S2 Table. Yeast Strains used in this study.**

| **Strain code** | **Name** | **Genotype** | **Origin** |
| --- | --- | --- | --- |
| BY4742 | WT | *MATα his3Δ1 leu2Δ0 lys2Δ0 ura3Δ0* | Euroscarf |
| Y15998 | *nup133Δ* | *MATα his3Δ1 leu2Δ0 lys2Δ0 ura3Δ0 nup133Δ::KanMx* | Euroscarf |
| Y14906 | *nup120Δ* | *MATα his3Δ1 leu2Δ0 lys2Δ0 ura3Δ0 nup120Δ::KanMx* | Euroscarf |
| ORD9803-10B | *nup84Δ* | *MATα his3Δ1 leu2Δ0 met15Δ0 ura3Δ0 nup84Δ::KanMx* | A gift from A. NICOLAS |
| Y17551 | *seh1Δ* | *MATα his3Δ1 leu2Δ0 lys2Δ0 ura3Δ0 seh1Δ::KanMx* | Euroscarf |
| YEF457 | *nup145ΔC* | *MATα ura3-52 leu2∆1 his3∆200 lys2∆202 nup145∆C::KanMx* | A gift from E. FABRE |
| yAMA195 | Ty1 BL-*lacZ* | *MATα flo8-1 ura3Δ851 trp1Δ63 his3Δ200 Ty1(BL)-lacZ-URA3* | [1] |
| yAMA159 | Ty1 BL-*lacZ nup133Δ* | *MATα flo8-1 ura3Δ851 trp1Δ63 his3Δ200 Ty1(BL)-lacZ-URA3 nup133Δ::KanMx* | This study^a^ |
| yAMA174 | Ty1 BL-*lacZ nup84Δ* | *MATα flo8-1 ura3Δ851 trp1Δ63 his3Δ200 Ty1(BL)-lacZ-URA3 nup84Δ::KanMx* | This study^b^ |
| yAMA196 | Ty1 BR-*lacZ* | *MATα flo8-1 ura3Δ851 trp1Δ63 his3Δ200 Ty1(BR)-lacZ-URA3* | [1] |
| yAMA161 | Ty1 BR-*lacZ nup133Δ* | *MATα flo8-1 ura3Δ851 trp1Δ63 his3Δ200 Ty1(BR)-lacZ-URA3 nup133Δ::KanMx* | This study^a^ |
| yAMA176 | Ty1 BR-*lacZ nup84Δ* | *MATα flo8-1 ura3Δ851 trp1Δ63 his3Δ200 Ty1(BR)-lacZ-URA3 nup84Δ::KanMx* | This study^b^ |
| yAMA206 | Ty1 DR1-*lacZ* | *MATα flo8-1 ura3Δ851 trp1Δ63 his3Δ200 Ty1(DR1)-lacZ-URA3* | [1] |
| yAMA163 | Ty1 DR1-*lacZ nup133Δ* | *MATα flo8-1 ura3Δ851 trp1Δ63 his3Δ200 Ty1(DR1)-lacZ-URA3 nup133Δ::KanMx* | This study^a^ |
| yAMA178 | Ty1 DR1-*lacZ nup84Δ* | *MATα flo8-1 ura3Δ851 trp1Δ63 his3Δ200 Ty1(DR1)-lacZ-URA3 nup84Δ::KanMx* | This study^b^ |
| yAMA208 | Ty1 DR4-*lacZ* | *MATα flo8-1 ura3Δ851 trp1Δ63 his3Δ200 Ty1(DR4)-lacZ-URA3* | [1] |
| yAMA167 | Ty1 DR4-*lacZ* *nup133Δ* | *MATα flo8-1 ura3Δ851 trp1Δ63 his3Δ200 Ty1(DR4)-lacZ-URA3 nup133Δ::KanMx* | This study^a^ |
| yAMA182 | Ty1 DR4-*lacZ* *nup84Δ* | *MATα flo8-1 ura3Δ851 trp1Δ63 his3Δ200 Ty1(DR4)-lacZ-URA3 nup84Δ::KanMx* | This study^b^ |
| yAMA209 | Ty1 DR6-*lacZ* | *MATα flo8-1 ura3Δ851 trp1Δ63 his3Δ200 Ty1(DR6)-lacZ-URA3* | [1] |
| yAMA169 | Ty1 DR6-*lacZ* *nup133Δ* | *MATα flo8-1 ura3Δ851 trp1Δ63 his3Δ200 Ty1(DR6)-lacZ-URA3 nup133Δ::KanMx* | This study^a^ |
| yAMA184 | Ty1 DR6-*lacZ* *nup84Δ* | *MATα flo8-1 ura3Δ851 trp1Δ63 his3Δ200 Ty1(DR6)-lacZ-URA3 nup84Δ::KanMx* | This study^b^ |
| yAMA197 | Ty1 LR1-*lacZ* | *MATα flo8-1 ura3Δ851 trp1Δ63 his3Δ200 Ty1(LR1)-lacZ-URA3* | [1] |
| yAMA170 | Ty1 LR1-*lacZ* *nup133Δ* | *MATα flo8-1 ura3Δ851 trp1Δ63 his3Δ200 Ty1(LR1)-lacZ-URA3 nup133Δ::KanMx* | This study^a^ |
| yAMA186 | Ty1 LR1-*lacZ* *nup84Δ* | *MATα flo8-1 ura3Δ851 trp1Δ63 his3Δ200 Ty1(LR1)-lacZ-URA3 nup84Δ::KanMx* | This study^b^ |
| yAMA198 | Ty1 LR4-*lacZ* | *MATα flo8-1 ura3Δ851 trp1Δ63 his3Δ200 Ty1(LR4)-lacZ-URA3* | [1] |
| yAMA59 | *spt3Δ* | *MATα his3Δ1 leu2Δ0 lys2Δ0 ura3Δ0 spt3Δ::HphMx* | This study^c^ |
| yAMA9 | *nup170Δ* | *MATα his3Δ1 leu2Δ0 lys2Δ0 ura3Δ0 nup170Δ::KanMx* | Euroscarf |
| Y15244 | *nup2Δ* | *MATα his3Δ1 leu2Δ0 lys2Δ0 ura3Δ0 nup2Δ::KanMx* | Euroscarf |
| Y14917 | *nup100Δ* | *MATα his3Δ1 leu2Δ0 lys2Δ0 ura3Δ0 nup100Δ::KanMx* | Euroscarf |
| Y16503 | *nup188Δ* | *MATα his3Δ1 leu2Δ0 lys2Δ0 ura3Δ0 nup188Δ::KanMx* | Euroscarf |
| YV1542 | *sus1Δ* | *MATα his3Δ1 leu2Δ0 lys2Δ0 ura3Δ0 sus1::KanMx* | [2] |
| YV1262 | *ulp1* | *MATα his3Δ1 leu2Δ0 met15Δ0 ura3Δ0 ulp1Δ::KanMx + YCpLac11-LEU2-ulp1-333* | [3] |
| YV553 | *mlp1Δ* | *MATα his3Δ1 leu2Δ0 lys2Δ0 ura3Δ0 mlp1Δ::KanMx* | Euroscarf |
| YV737 | *mlp2Δ* | *MATa his3Δ1 leu2Δ0 met15Δ0 ura3Δ0 mlp2∆::KanMx* | Euroscarf |
| yAMA17 | *mlp1Δ mlp2Δ* | *MAT? his3Δ1 leu2Δ0 lys2Δ0 ura3Δ0 mlp1Δ::KanMx mlp2Δ::KanMx* | This study^d^ |
| YV1410 | *ubc9-1* | *MATa his3Δ1 leu2Δ0 met15Δ0 ura3Δ0 ubc9∆::KanMx + pRS315-ubc9-1* | [4] |
| YV1478 | *siz1Δ siz2Δ* | *MATa his3Δ1 leu2Δ0 lys2Δ0 ura3Δ0 siz1Δ::KanMx siz2Δ::KanMx* | [3] |
| yAMA61 | *ΔN-ulp1* | *MATα his3Δ1 leu2Δ0 lys2Δ0 ura3Δ0 ∆N338-ulp1::HIS3* | This study^e^ |
| yAMA77 | *ΔN-ulp1nup133Δ* | *MATα his3Δ1 leu2Δ0 lys2Δ0 ura3Δ0 ∆N338-ulp1::HIS3 nup133Δ::KanMx* | This study^a^ |
| yAMA226 | *ΔN-ulp1nup84Δ* | *MATα his3Δ1 leu2Δ0 lys2Δ0 ura3Δ0 ∆N338-ulp1::HIS3 nup84Δ::KanMx* | This study^b^ |
| yAMA70 | *ΔN-ulp1 his-* | *MATα his3Δ1 leu2Δ0 lys2Δ0 ura3Δ0 ∆N338-ulp1::his3::LYS2* | This study^f^ |
| yAMA60 | *adk1Δ* | *MATα his3Δ1 leu2Δ0 lys2Δ0 ura3Δ0 adk1Δ::HphMx* | This study^g^ |
| yAMA229 | *com2Δ* | *MATa his3Δ1 leu2Δ0 met15Δ0 ura3Δ0 com2∆::KanMx* | Euroscarf |
| yAMA230 | *mig2Δ* | *MATa his3Δ1 leu2Δ0 met15Δ0 ura3Δ0 mig2∆::KanMx* | Euroscarf |
| yAMA231 | *rof1Δ* | *MATa his3Δ1 leu2Δ0 met15Δ0 ura3Δ0 rof1∆::KanMx* | Euroscarf |
| yAMA232 | *yor338wΔ* | *MATa his3Δ1 leu2Δ0 met15Δ0 ura3Δ0 yor338w∆::KanMx* | Euroscarf |
| yAMA233 | *usv1Δ* | *MATa his3Δ1 leu2Δ0 met15Δ0 ura3Δ0 usv1∆::KanMx* | Euroscarf |
| yAMA234 | *gsp2Δ* | *MATa his3Δ1 leu2Δ0 met15Δ0 ura3Δ0 gsp2∆::KanMx* | Euroscarf |
| yAMA235 | *rim4Δ* | *MATa his3Δ1 leu2Δ0 met15Δ0 ura3Δ0 rim4∆::KanMx* | Euroscarf |
| yAMA236 | *tis11Δ* | *MATa his3Δ1 leu2Δ0 met15Δ0 ura3Δ0 tis11∆::KanMx* | Euroscarf |
| yAMA247 | *xbp1Δ* | *MATa his3Δ1 leu2Δ0 met15Δ0 ura3Δ0 xbp1∆::KanMx* | Euroscarf |
| JC3787 | Ty1*his3AI*-3114 | *MATα his3Δ1 leu2Δ0 lys2Δ0 ura3Δ0*  *Ty1his3AI[Δ1]-3114* | [5] |
| yAMA141 | Ty1*his3AI*-3114 *nup133Δ* | *MATα his3Δ1 leu2Δ0 lys2Δ0 ura3Δ0*  *Ty1his3AI[Δ1]-3114 nup133Δ::KanMx* | This study^a^ |
| yAMA147 | Ty1*his3AI*-3114 *nup120Δ* | *MATα his3Δ1 leu2Δ0 lys2Δ0 ura3Δ0*  *Ty1his3AI[Δ1]-3114 nup120Δ::KanMx* | This study^h^ |
| yAMA143 | Ty1*his3AI*-3114 *nup84Δ* | *MATα his3Δ1 leu2Δ0 lys2Δ0 ura3Δ0*  *Ty1his3AI[Δ1]-3114 nup84Δ::KanMx* | This study^b^ |
| yAMA149 | Ty1*his3AI*-3114 *seh1Δ* | *MATα his3Δ1 leu2Δ0 lys2Δ0 ura3Δ0*  *Ty1his3AI[Δ1]-3114 seh1Δ::KanMx* | This study^i^ |
| yAMA151 | Ty1*his3AI*-3114 *nup145ΔC* | *MATα his3Δ1 leu2Δ0 lys2Δ0 ura3Δ0*  *Ty1his3AI[Δ1]-3114 nup145∆C::KanMx* | This study^j^ |
| yAMA199 | Ty1*his3AI*-3114 *spt3Δ* | *MATα his3Δ1 leu2Δ0 lys2Δ0 ura3Δ0*  *Ty1his3AI[Δ1]-3114 spt3∆::HphMx* | This study^c^ |
| yAMA205 | Ty1*his3AI*-3114 *ΔN-ulp1* | *MATα his3Δ1 leu2Δ0 lys2Δ0 ura3Δ0*  *Ty1his3AI[Δ1]-3114 ∆N338-ulp1::his3::LYS2* | This study^k^ |
| yAMA201 | Ty1*his3AI*-3114 *adk1Δ* | *MATα his3Δ1 leu2Δ0 lys2Δ0 ura3Δ0*  *Ty1his3AI[Δ1]-3114 adk1∆::HphMx* | This study^l^ |
| yAMA203 | Ty1*his3AI*-3114 *fus3Δ* | *MATα his3Δ1 leu2Δ0 lys2Δ0 ura3Δ0*  *Ty1his3AI[Δ1]-3114 fus3∆::HphMx* | This study^m^ |
| LV1755 | Ty1*his3AI*-3114 *rrm3Δ* | *MATα his3Δ1 leu2Δ0 lys2Δ0 ura3Δ0*  *Ty1his3AI[Δ1]-3114 rrm3∆::KanMx* | This study^n^ |
| yAMA227 | Ty1*his3AI*-3114 *mlp1Δ* | *MATα his3Δ1 leu2Δ0 lys2Δ0 ura3Δ0*  *Ty1his3AI[Δ1]-3114 mlp1∆::KanMx* | This study^o^ |
| yAMA239 | Ty1*his3AI*-3114 *mlp1Δ mlp2Δ* | *MAT? his3Δ1 leu2Δ0 lys2? met15 ? ura3Δ0 Ty1his3AI[Δ1]-3114 mlp1∆::KanMx mlp2∆::KanMx* | This study^p^ |
| DG2122 | pBJC573/Ty1*his3-AI* | *MATα his3Δ1 leu2Δ0 lys2Δ0 ura3Δ0*  *pBJC573/Ty1his3-AI* | [6] |
| yAMA248 | pBJC573/Ty1*his3-AI nup133Δ* | *MATα his3Δ1 leu2Δ0 lys2Δ0 ura3Δ0*  *pBJC573/Ty1his3-AI nup133Δ::KanMx* | This study^a^ |
| yAMA250 | pBJC573/Ty1*his3-AI nup84Δ* | *MATα his3Δ1 leu2Δ0 lys2Δ0 ura3Δ0*  *pBJC573/Ty1his3-AI nup84Δ::KanMx* | This study^b^ |

a. *NUP133* complete CDS was deleted by a KanMX cassette amplified from the gDNA of yAMA1.

b. *NUP84* complete CDS was deleted by a KanMX cassette amplified from pFA6a-KanMX6.

c. *SPT3* complete CDS was deleted by a HphMX cassette amplified from pAG32.

d. Segregant of diploids constructed from YV553 & YV737.

e. Integration of the *∆N338-ulp1* allele within BY4742 using pCR4Blunt-ΔN-ULP1-HIS3.

f. The original *HIS3* marker was swapped by homologous recombination with a *his3::LYS2* disruption fragment.

g. *ADK1* complete CDS was deleted by a HphMX cassette amplified from pAG32.

h. *NUP120* complete CDS was deleted by a KanMX cassette amplified from the gDNA of yAMA2.

i. *SEH1* complete CDS was deleted by a KanMX cassette amplified from the gDNA of yAMA52.

j. The region of *NUP145* encoding its C-ter domain was deleted by a KanMX cassette amplified from the gDNA of yAMA53.

k. The *∆N338-ulp1* allele was integrated using a *∆N338-ulp1::LYS2* cassette amplified from the gDNA of yAMA70.

l. *ADK1* complete CDS was deleted by a HphMX cassette amplified from the gDNA of yAMA60.

m. *FUS3* complete CDS was deleted by a HphMX cassette amplified from pFA6a-HphMX6.

n. *RRM3* complete CDS was deleted by a KanMX cassette amplified from pFA6a-KanMX6 (provided by A. Asif-Laidin in the Lesage lab).

o. *MLP1* complete CDS was deleted by a KanMX cassette amplified from the gDNA of yAMA17.

p. Segregant of diploids constructed from yAMA227 & YV737.

**REFERENCES**

1. Morillon A, Bénard L, Springer M, *et al.* Differential Effects of Chromatin and Gcn4 on the 50-Fold Range of Expression among Individual Yeast Ty1 Retrotransposons. *Mol. Cell. Biol.* 2002 ; 22 : 2078–2088.

2. Bonnet A, Bretes H, Palancade B. Nuclear pore components affect distinct stages of intron-containing gene expression. *Nucleic Acids Res.* 2015 ; 43 : 4249–4261.

3. Bretes H, Rouviere JO, Leger T, *et al.* Sumoylation of the THO complex regulates the biogenesis of a subset of mRNPs. *Nucleic Acids Res.* 2014 ; 42 : 5043–58.

4. Rouvière JO, Bulfoni M, Tuck A, *et al.* A SUMO-dependent feedback loop senses and controls the biogenesis of nuclear pore subunits. *Nat. Commun.* 2018 ; 9 : 1665.

5. Mou Z, Kenny AE, Curcio MJ. Hos2 and Set3 promote integration of Ty1 retrotransposons at tRNA genes in Saccharomyces cerevisiae. *Genetics* 2006 ; 172 : 2157–2167.

6. Nyswaner KM, Checkley MA, Yi M, *et al.* Chromatin-associated genes protect the yeast genome from Ty1 insertional mutagenesis. *Genetics* 2008 ; 178 : 197–214.
