# Supplementary material for: A nuclear pore sub-complex restricts the propagation of Ty retrotransposons by limiting their transcription
Source: PLoS Genet. 2021 Nov 1;17(11):e1009889. doi: 10.1371/journal.pgen.1009889 (PMC8585004; doi:10.1371/journal.pgen.1009889)
Supplement: S3 Table — (DOCX) [file pgen.1009889.s008.docx]

**S3 Table. Plasmids used in this study.**

| **Name** | **Description** | **Origin** |
| --- | --- | --- |
| pFA6a-KanMx6 | for deletion | [1] |
| pFA6a-HphMx6 | for deletion | [2] |
| pAG32 | for deletion | [3] |
| pCR4Blunt-ΔN-ULP1-HIS3 | for integration of the *∆N-ulp1::HIS3* allele at the *ULP1* locus | [4] |
| D1433 | his3::LYS2 disruption fragment | [5] |
| pUN100-protA-NUP133 | CEN/AmpR/LEU2/proteinA-Nup133 | [6] |
| pUN100-protA-ΔN-NUP133 | CEN/AmpR/LEU2/proteinA-ΔN-Nup133 | [6] |
| pCenTy | CEN/AmpR/URA3/Ty1-*his3AI* | [7] |
| pGal-Ty1 | 2µ/AmpR/URA3/pGAL1-Ty1-*his3AI* | [8] |

**REFERENCES**

1. Longtine MS, McKenzie A, Demarini DJ, *et al.* Additional modules for versatile and economical PCR-based gene deletion and modification in Saccharomyces cerevisiae. *Yeast* 1998 ; 14 : 953–961.

2. Hentges P, Driessche B Van, Tafforeau L, *et al.* Three novel antibiotic marker cassettes for gene disruption and marker switching in Schizosaccharomyces pombe. *Yeast* 2005 ; 22 : 1013–1019.

3. Goldstein AL, McCusker JH. Three new dominant drug resistance cassettes for gene disruption in Saccharomyces cerevisiae. *Yeast* 1999 ; 15 : 1541–1553.

4. Palancade B, Liu X, Garcia-Rubio M, *et al.* Nucleoporins prevent DNA damage accumulation by modulating Ulp1-dependent sumoylation processes. *Mol. Biol. Cell* 2007 ; 18 : 2912–23.

5. Voth WP, Jiang YW, Stillman DJ. New “marker swap” plasmids for converting selectable markers on budding yeast gene disruptions and plasmids. *Yeast* 2003 ; 20 : 985–993.

6. Doye V, Wepf R, Hurt EC. A novel nuclear pore protein Nup133p with distinct roles in poly(A)+ RNA transport and nuclear pore distribution. *EMBO J.* 1994 ; 13 : 6062–6075.

7. Salinero AC, Knoll ER, Zhu ZI, *et al.* The Mediator co-activator complex regulates Ty1 retromobility by controlling the balance between Ty1i and Ty1 promoters. *PLOS Genet.* 2018 ; 14 : e1007232.

8. Curcio MJ, Garfinkel DJ. Single-step selection for Ty1 element retrotransposition. *Proc. Natl. Acad. Sci. U. S. A.* 1991 ; 88 : 936–40.
