# Supplementary material for: A nuclear pore sub-complex restricts the propagation of Ty retrotransposons by limiting their transcription
Source: PLoS Genet. 2021 Nov 1;17(11):e1009889. doi: 10.1371/journal.pgen.1009889 (PMC8585004; doi:10.1371/journal.pgen.1009889)
Supplement: S4 Table — (DOCX) [file pgen.1009889.s009.docx]

**S4 Table. Primers used in this study.**

| **Name** | **Sequence** |
| --- | --- |
| O-AMA8_Ty1-midF | cattgcgtcaaatgagatccaa |
| O-AMA9_Ty1-midR | ggtgtggaatcggttggactc |
| O-AMA18_Ty2-midF | GTGATACAAATATCAAATACAGGAC |
| O-AMA19_Ty2-midR | CAGCCTTTGTTTCTATGGAGA |
| O-AMA285_Ty3-midF | ACTTGTTGGTGTCGTCGGTT |
| O-AMA286_Ty3-midR | CGGAAGTGGTGGAGTGCTTT |
| O-AMA14_Ty1-5'2F | tggaacgcctctgagcactc |
| O-AMA15_Ty1-5'2R | cattaggtgaggttaacattg |
| O-AMA76_LacZ5'-F | ttcctgaggccgatactgtc |
| O-AMA77_LacZ5'-R | tgggataggttacgttggtg |
| O-AMA10_Act1F | ACGTTACCCAATTGAACACG |
| O-AMA11_Act1R | AGAACAGGGTGTTCTTCTGG |
| O-AMA127_PMA1-midF | TTGCCAGCTGTCGTTACCAC |
| O-AMA128_PMA1-midR | TCGACACCAGCCAAGGATTC |
| O-AMA269_intergenic #1-F | GAAACCACGAAAAGTTCACCA |
| O-AMA270_intergenic #1-R | AGCTTCTGCAAACCTCATTTG |
| O-AMA36_Ty1-His3F | TGTGATGACAAAACCTCTTCCG |
| O-AMA37_Ty1-His3R | ACGATGTTCCCTCCACCAAA |
| O-AMA158_Adapteur F | GTAATACGACTCACTATAGGGCACGCGTGGTCGACGGCCCGGGCTGGT |
| O-AMA159_Adapteur R | ACCAGCCC |
| O-AMA178_LTR_F | gcctttatcaacaatggaatcccaac |
| O-AMA239_chrI_MssI81808F | GCCATTATTTTGTGAGTTCTGG |
| O-AMA243_chXIVMssI346731F | AAGTGGGGATGAATTTGCTG |
| O-AMA156_P278 | ACTATAGGGCACGCGTGGT |
| O-AMA340_intergenic #2-F | CGCATTACCAGACGGAGATGT |
| O-AMA341_intergenic #2-R | CAAGCAAGCCTTGTGCATAAGA |
| O-AMA344_PSP2_5’F | AGCTTGACTCACCAGCTCTT |
| O-AMA345_PSP_3’R | TATCGTCGCCACCTTTCTCA |
